# Physicochemical Characterization and Structural Tuning of PVA/CMC Composites Modified with Montmorillonite and Glycerol: Toward Sustainable Wound Dressings

**DOI:** 10.3390/ijms262311445

**Published:** 2025-11-26

**Authors:** Aleksandra Niedźwiecka, Irena Brunarska, Anna Adamczyk, Przemysław Talik

**Affiliations:** 1 Faculty of Chemistry and Pharmacy, University of Opole, Oleska 48, 45-052 Opole, Poland; aleksandra.niedzwiecka@uni.opole.pl; 2 Institute of Geological Sciences, Jagiellonian University, Gronostajowa 3a, 30-387 Krakow, Poland; irena.brunarska@uj.edu.pl; 3 Faculty of Materials Science and Ceramics, AGH University of Krakow, Av. Mickiewicza 30, 30-059 Krakow, Poland; aadamcz@agh.edu.pl; 4 Faculty of Pharmacy, Jagiellonian University Medical College, 9 Medyczna St., 30-688 Krakow, Poland

**Keywords:** carboxymethyl cellulose (CMC), carboxymethyl cellulose-poly(vinyl alcohol) hydrogels, modified composites, montmorillonite

## Abstract

Current wound care still lacks multifunctional dressings capable of providing both structural support and controlled therapeutic activity. Developing such materials requires polymer matrices with tunable physicochemical properties, achievable through the rational selection of components and modifiers. In this study, polymer composites based on poly(vinyl alcohol) (PVA) and carboxymethyl cellulose (CMC), modified with glycerol and montmorillonite (MMT), were synthesized using a novel and rapid preparation method aimed at producing structurally adjustable matrices. The obtained materials were characterized by FTIR, SEM, and AFM analyses. FTIR spectra confirmed the competitive role of glycerol in reducing cross-linking efficiency and the compensatory effect of MMT in enhancing molecular interactions and structural density. The interaction between the two modifiers resulted in hybrid structures with intermediate organization, neither purely crystalline nor amorphous. SEM imaging revealed that glycerol promotes porosity, while higher MMT content stabilizes the polymer matrix, enhancing surface homogeneity. AFM revealed that the average roughness (1 × 1 μm scans) increased from 0.98 μm (sample B7) to 5.30 μm (sample C7) after glycerol addition. In conclusion, obtained results demonstrate the synergistic and opposing roles of glycerol and MMT, ultimately enabling the design of multifunctional polymer composites and warranting further investigation through XRD, DSC, rheology, and swelling capacity analyses.

## 1. Introduction

Dressing materials play an important role in the wound healing process. They provide a physical barrier protecting tissues from infection and mechanical damage and absorb exudate. Traditional dressings, such as cotton wool or gauze, are often used in clinical practice because they are economical. Unfortunately, their fibers stick to the granulation tissue, causing its damage and pain when removing or changing the dressing [1]. Modern dressings such as hydrocolloids, alginates, hydrogels, foams, and films cause less pain during dressing changes and ensure proper wound temperature and moisture, thereby promoting fibroblast proliferation, accelerating the reepithelialization process, and reducing overall discomfort [1,2,3,4]. Modern wound dressings may also contain pharmacologically active substances such as antibiotics, non-steroidal anti-inflammatory drugs, analgesics, and local anesthetics or natural extracts with anti-inflammatory, antioxidant, and antimicrobial properties [1,3,4].

Recent research highlights a wide range of hydrogel-based dressings formulated from natural and/or synthetic polymer matrices. Natural polymer hydrogels, such as chitosan, alginate, hyaluronic acid, and cellulose derivatives, are widely studied due to their biocompatibility, biodegradability, and intrinsic wound-healing. In contrast, synthetic hydrogels based on, e.g., poly(vinyl alcohol) (PVA), polyethylene glycol (PEG), or polyurethane (PU) provide superior mechanical strength and structural stability, making them suitable for load bearing or highly exuding wounds [5,6]. Emerging composite hydrogels combine these two classes to overcome individual limitations and introduce multifunctional behavior—for example, enhanced swelling, drug-loading capacity, and antibacterial performance. These advances demonstrate a clear trend toward tailoring polymer networks and incorporating functional additives to optimize moisture management, mechanical robustness, therapeutic delivery, etc., in modern wound dressings [7,8].

The ideal dressing must have the capacity to adequately absorb exudate. Both excess and insufficient exudate impede healing: too much exudate causes maceration of the skin and damage to adjacent wound areas, whereas too little exudate dehydrates the wound and impairs cell migration [1]. Gas exchange is also important. The ideal dressing must also have adequate gas permeability. If the permeability index is too high, the wound becomes dry, while if it is low, excessive exudation can lead to wound infection. In addition, non-toxicity, tissue compatibility, sterility, and antimicrobial activity should not be overlooked. Adequate mechanical strength in terms of changes in stiffness, elasticity, and resilience (adapting to a given phase of wound healing), an adequate rate of degradation consistent with the rate of healing, ease of use including trouble-free removal after healing, and economy of use are also desirable [1,2,3,4,9,10]. Although the ideal dressing does not exist, with appropriate modifications, it is possible to obtain countless combinations of dressing/matrix properties.

A carefully conducted search was undertaken to identify components that would meet the above conditions and provide the material with absorbent properties, sufficient mechanical strength, appropriate adhesion to the wound site, and the capacity to incorporate therapeutic agents and control their release. The goal was to develop a composite with excellent swelling capacity, appropriate material hydration, biocompatibility, and biodegradability that could be further modified—for example, by increasing pore volume and surface area, cation exchange, or interlayer intercalation. Therefore, carboxymethyl cellulose, poly(vinyl alcohol), montmorillonite, glycerin, and citric acid were selected as the components of synthesized composites.

Carboxymethyl cellulose (CMC) is a semi-synthetic cellulose derivative that is readily used in many industries due to its relatively low production cost as well as its many application possibilities due to its properties such as excellent swelling capacity, pH sensitivity, biocompatibility, and biodegradability. Although it is a special material in the context of producing hydrogels for different applications, including wound dressing, tissue engineering, and drug delivery matrices, it adapts easily to irregularly shaped wounds, maintains a moist wound environment, and promotes autolytic debridement. CMC has been shown to enhance wound healing in in vivo models [1]. Furthermore, it effectively regulates transepidermal water loss and exhibits significant exudate absorption capacity, which minimizes moisture loss in the wound microenvironment. However, due to its low mechanical strength and lack of specific antimicrobial activity, CMC is included in composites rather than being utilized as a dressing material on its own. Its chemical structure allows for functionalization enabling the development of composites with enhanced and targeted properties [1,9].

Poly(vinyl alcohol) (PVA) is a vinyl polymer obtained by hydrolysis of poly(vinyl acetate). It makes a composite more hydrated and increases its mechanical resistance and thermal stability. It promotes cell adhesion and migration, making it a good component of hydrogel composites. It is environmentally friendly, and the interactions of CMC with PVA are mainly based on hydrogen bonds between the carboxyl groups of CMC and the hydroxyl groups of PVA. They are miscible and compatible. However, it does not show antimicrobial activity on its own, nor does it directly accelerate wound healing, which is why, among other reasons, it is not found as a stand-alone dressing agent but is included in composites [11,12].

Another substrate of significant synthetic interest is montmorillonite (MMT). It has been used to modify both the mechanical and structural properties of the composite, as it can, among other effects, increase the pore volume and surface area. It is derived from bentonite and has the general formula: (Al_2_-_x_Mg_x_)Si_4_O_10_(OH)_2_×(M×nH_2_O) (M: Na^+^, Ca^2+^, and Mg^2+^, etc.) [13]. It has a layered structure and is made up of negatively charged packets balanced by cations (sodium, calcium, lithium, aluminum, or magnesium) between the packets. Each of these is characterized by a typical 2:1 structure for silicates, i.e., two tetrahedral layers made up of silicon atoms, although aluminum atoms are possible, and one octahedral layer made up of aluminum atoms, although silicon, magnesium, or iron atoms are also possible. The composition of MMT varies depending on the site of extraction and the layer of the deposit. Montmorillonite has the ability to absorb water. This occurs by surface adsorption and because it has inter-pack water layers by absorption of water molecules, which manifests itself by swelling, i.e., a change in lattice spacing [1,13,14,15,16]. It has siloxane groupings, contributing a negative charge and imparting weak alkaline properties, and originating from Si-O-Si and Al-O-Al bonds broken at the edge of the mineral particles, which can transform into groups: Si-OH and Al-OH. This affects, e.g., the acid–base properties and is important in terms of the formation of hydrogen bonds between the components of the composite and thus changes its physicochemical properties [14,15,16,17].

Citric acid (CA) was selected as a cross-linking agent. It has good biocompatibility and low toxicity. Hydrogels are completely safe during synthesis and have good swelling and biodegradation properties. When cross-linking is carried out under appropriate conditions, esterification reactions take place. Citric acid with its three carboxyl functional groups can interact with hydroxyl groups of the polymer chains, leading to the formation of cross-links between the chains. Excess residual-free CA can work as a plasticizer, resulting in the enhancement of polymer dispersion [18,19,20].

And the last ingredient (of matrix) is glycerin, chosen as a plasticizer to decrease brittleness and enhance ductility. However, the intention of usage of large amounts of plasticizer (glycerol and residual CA) was to decrease resistance and improve adhesiveness [19].

To summarize, the following materials were selected:

- carboxymethyl cellulose because it provides excellent swelling capacity, pH sensitivity, biocompatibility, and biodegradability;
- poly(vinyl alcohol) because it enhances the hydration of composite materials and improves their mechanical strength and thermal stability. It also promotes cell adhesion and migration, making it a valuable component in hydrogel composites for biomedical applications;
- montmorillonite because it is used to modify the mechanical and structural properties of the composite. Due to its layered silicate structure and high aspect ratio, MMT restricts polymer chain mobility and increases structural rigidity. It can also increase pore volume and surface area. MMT contributes to enhanced molecular adhesion through multiple interfacial interactions. Its surface contains Si-OH and Al-OH groups and negatively charged silicate layers that can establish hydrogen bonding, electrostatic interactions, and van der Waals forces with the functional groups of the polymer chains (e.g., hydroxyl or carboxyl groups in PVA and CMC). Additionally, exfoliated MMT exposes an additional active surface area. This facilitates stronger adhesion not only between polymer chains but also between the composite and active molecules, improving the potential for molecular entrapment/adsorption or drug-loading efficiency in biomedical systems. Due to its layered structure—composed of negatively charged packets balanced by cations between the packets—it enables further modifications such as cation exchange and interlayer intercalation [14,15,16,17];
- glycerin as a plasticizer to reduce polymer rigidity, decrease brittleness, and enhance ductility;
- citric acid because hydrogels are completely safe during synthesis and have good swelling and biodegradation properties, while also reducing resistance and improving the material’s adhesiveness.

The intended properties of the composites (matrices) were evaluated using a range of analytical techniques. Fourier transform infrared (FTIR) spectroscopy was employed to assess the chemical structure and degree of cross-linking. Scanning electron microscopy (SEM) was used to examine the homogeneity of the composites, the dispersion of montmorillonite, and the presence of pores or structural features potentially suitable for future drug incorporation. Atomic force microscopy (AFM) provided topographic and phase images, enabling precise determination of surface roughness and further characterization of the surface morphology.

Current wound care still lacks multifunctional dressings capable of simultaneously providing structural support, antimicrobial activity, controlled drug release, and tissue regeneration stimulation. Taking all the above into consideration, the aim of this study was to develop sustainable PVA/CMC-based composites with tunable properties, achieved through the addition of montmorillonite and glycerol, for future potential use as wound dressing materials and drug-release matrices. Particular emphasis was placed on structure–property control, allowing adjustment of the composites’ functional characteristics. To the best of the authors’ knowledge, the applied synthesis route was novel in terms of both its short duration and the sequence of preparation steps.

## 2. Results and Discussion

### 2.1. Preparation of Composite Materials

**Figure 1:**
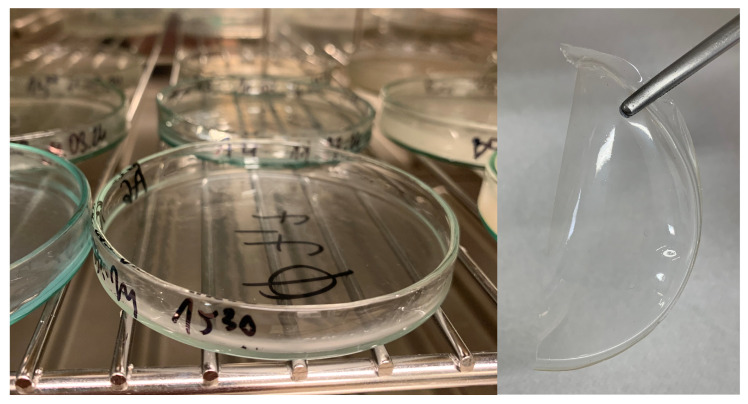
Photographs of the composites during the drying process after pouring them into Petri dishes (**left**) and of the fully dried film (**right**).

The synthesis method was novel in terms of both duration (quick synthesis) and the sequence of composite preparation. The materials synthesized (Figure 1) using this method exhibited appropriate mechanical (in terms of brittleness and ductility) behavior. Composites A2, A3, C5, and C7 (with glycerin) were more ductile than A1, B5, and B7 (without glycerin); thus, composites A1, B5, and B7 were more brittle than composites A2, A3, C5, and C7.

### 2.2. Attenuated Total Reflectance-Fourier Transform Infrared Spectroscopy (ATR-FTIR)

**Figure 2:**
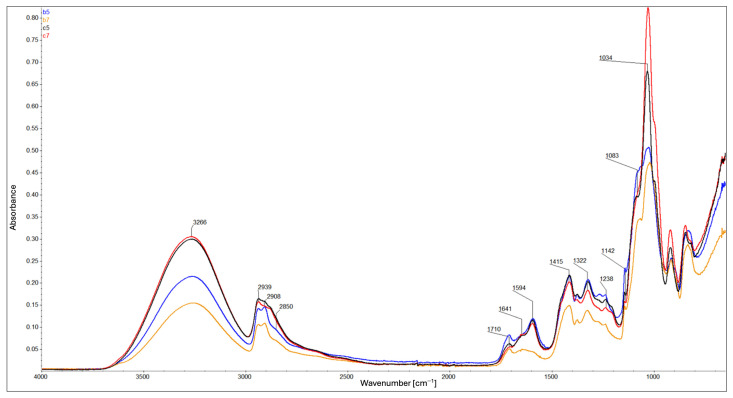
ATR-FTIR spectra of B5 (blue), B7 (yellow), C5 (black), C7 (red): composites with MMT.

**Figure 3:**
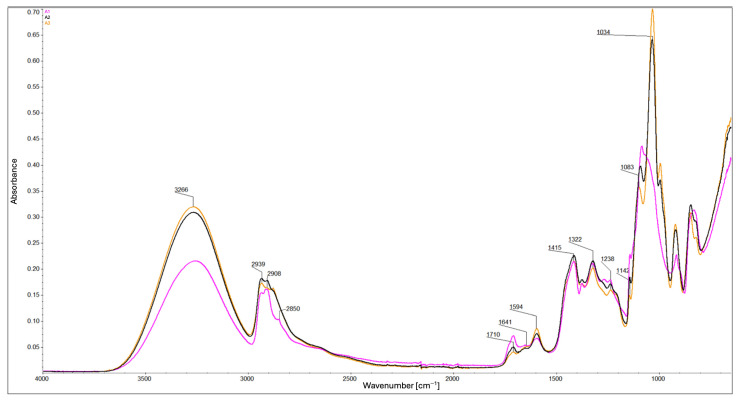
ATR-FTIR spectra of A1 (pink), A2 (black), and A3 (yellow): non-MMT composites.

**Figure 4:**
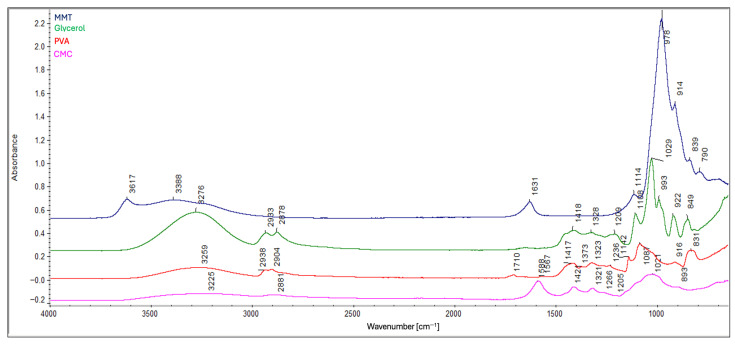
ATR-FTIR spectra of MMT (navy blue), glycerol (green), PVA (red), and CMC (pink).

FTIR analysis provided spectra for the composite samples: B5, B7, C5, C7 (Figure 2), A1, A2, and A3 (Figure 3), as well as for the individual components: carboxymethyl cellulose, polyvinyl alcohol, glycerin, and montmorillonite (Figure 4) for comparison. FTIR spectra were baseline-corrected using a common baseline region and normalized to an internal standard, defined as the sum of the relative peak heights at 2939 cm^−1^, 2908 cm^−1^, and 2850 cm^−1^ since the total content of –CH_2_ and –CH_3_ groups in the composite remains constant. The ratio of the absorbance of the selected analytical band to the internal standard band was used to evaluate relative changes in chemical structure.

In all of them, the following were identified: the band at 3266 cm^−1^ corresponds to the O–H stretching vibrations. The peaks at 2939 cm^−1^ and 2908 cm^−1^ are attributable to the asymmetric and symmetric stretching vibrations of the –CH_2_, respectively. The peak at 2850 cm^−1^ corresponds to the stretching vibrations of the –CH_3_. The peak at 1710 cm^−1^ is due to the stretching vibrations of the C=O bond in ester groups. The peaks at 1641 cm^−1^ and 1594 cm^−1^ are attributable to the asymmetric vibrations of the COO^−^ group. The peaks at 1415 cm^−1^ and 1322 cm^−1^ are due to symmetric vibrations of the COO^−^ group. The peak at 1238 cm^−1^ is also related to ester bonds. The peaks in the range of 1150–1000 cm^−1^ are due to the stretching vibrations of the C–O bond [11,18,21,22,23,24]. All of the peaks are listed in Table 1.

ATR-FTIR analysis revealed a relationship between glycerol content and values of the relative peak height ratios at 3266 cm^−1^ relative to the internal standard: higher glycerol content corresponds to a higher ratio, while lower glycerol content results in a lower ratio value. In composites B5 and B7, the effect of montmorillonite is evident—higher MMT content leads to decreased reflectance values. In composites C5 and C7 (Figure 2), this effect appears to be mitigated by the presence of glycerol. These observations indicate a limited number of –OH groups compared to composites with lower glycerol content. The higher ratio values observed in composites C5, C7, A2, and A3 are likely due to the addition of –OH-rich components, whereas B5, B7, and A1 show lower ratio values due to limited –OH availability [11,21,22] (Figure 2 and Figure 3). Addition of a higher concentration of MMT enhances molecular interactions within the matrix, increases structural density, and promotes hydrogen bond formation through available –OH groups. This effect is counteracted by the presence of glycerin, which acts as an intermediate element—it enables the formation of hydrogen bonds with each component of the composite, thereby separating the polymer chains from each other [11,25] (Figure 2 and Figure 3). Composites containing glycerol exhibit significantly lower ratio values at 2850 cm^−1^ compared to their non-glycerol counterparts, which show higher values. A higher ratio value observed at similar levels in composites C5 and C7 (Figure 2) indicates a higher content of methyl (–CH_3_) groups. Considering also the ratios at 2850 cm^−1^ to those at 2908 and 2939 cm^−1^, this trend may suggest polymer chain cleavage, resulting in a higher proportion of shorter chains and a reduced presence of longer polymer segments, as well as a spatial organization of the composite structure that is distinct from that of the other formulations. Similar trends are also observed in composites A2 and A3 (Figure 3), indicating that glycerol, rather than MMT, plays a key role in this process. For the peaks at 2908 cm^−1^ and 2939 cm^−1^, the values of the relative peak height ratios relative to the internal standard appear to correlate with glycerol content (Figure 2). Glycerol-containing composites show relatively consistent ratio values at these bands, whereas notable differences are observed among the non-glycerol composites. Lower glycerol content corresponds to higher ratio values at these wavenumbers (Figure 2 and Figure 3). These findings imply a higher concentration of methylene (–CH_2_–) groups in the glycerol-containing composites and a lower concentration in those without glycerol. Similar effects are also observed in composites A1 and A2 (Figure 3), further confirming the significant role of glycerol. This trend is further reflected at wavenumbers 1322 cm^−1^ and 1415 cm^−1^, where composites B5 and B7 exhibit the highest ratio values among all samples. The remaining composites show relatively similar values, reinforcing the observation that cross-linking efficiency and molecular packing are strongly influenced by the presence and quantity of glycerol. Based on the values of the relative peak height ratios at wavenumbers 1594 cm^−1^ (closely associated with the degree of CMC substitution) and 1710 cm^−1^ relative to the internal standard, it was determined that the protonated form of the carboxyl group (–COOH) predominates only in composites A1 and B7 (Figure 2 and Figure 3), whereas in the remaining composites, the deprotonated form (–COO^−^) is dominant [11]. In the presence of glycerol, the increased consumption of –OH groups during esterification (i.e., the polycondensation reaction between glycerol and citric acid) may result in a local increase in pH, thereby promoting the deprotonation of carboxyl groups. This mechanism likely accounts for the predominance of the deprotonated form (–COO^−^) observed in glycerol-containing composites [26]. Moreover, competitive interactions between carboxyl group deprotonation and hydrogen bond formation with MMT may explain why composite B5 exhibits a higher proportion of deprotonated groups compared to B7. At elevated concentrations, MMT—owing to its content of exchangeable cations (e.g., Na^+^, Ca^2+^)—can function as a “proton trap”, thereby shifting the acid–base equilibrium toward deprotonation [13,14,15]. This also helps to explain the predominance of the protonated form (–COOH) in composite A1, where the absence of MMT (lack of ion-exchange sites capable of proton trapping) favors the retention of protons on the carboxyl groups. Analysis of the spectral region around 1710 cm^−1^ further revealed that composites without glycerol (A1) exhibit a higher ratio value, which is attributed to the greater extent of substitution of hydrogen bonds between PVA and CMC chains by ester bonds than their glycerol-containing counterparts (A2, A3; Figure 3). This indicates that glycerol content influences the efficiency of cross-linking [21]. In composites B5 and C5 (as compared to B7 and C7, respectively), the higher ratio values at 1710 cm^−1^ correspond to an increase in the number of C=O bonds, indicating increased cross-linking efficiency. Accordingly, composites B5 and C5 appear to be more effectively cross-linked than B7 and C7. When comparing composites A1, A2, B5, and C5 (Figure 2 and Figure 3), it is evident that the addition of glycerol reduces the cross-linking level of the composite, whereas the simultaneous addition of MMT mitigates this effect—as indicated by the higher ratio values for composite C5 compared with A2, but lower compared with A1. Likewise, the ratio value for composite B5 is higher than that for A1. Composites with higher glycerol content exhibit decreased ratio values at 1238 cm^−1^, which is associated with ester bond formation, reinforcing the conclusion that glycerol reduces cross-linking efficiency (Figure 3). Similarly, composites with lower MMT content appear to be more effectively cross-linked than those with higher MMT content, as evidenced by the formation of a greater number of ester bonds (Figure 2 and Figure 3). The peak at 1142 cm^−1^ corresponds to the crystalline phase, and the peak at 1083 cm^−1^ is attributed to the (C–O) stretching vibration as representative of the amorphous phase [24]. Values of the relative peak height ratios at 1083 cm^−1^ and 1034 cm^−1^ relative to the internal standard are higher for composite B5 than for C5. This indicates the highest proportion of the amorphous and crystalline phases in composite B5 than in C5. A similar pattern is observed at 1142 cm^−1^, where composite B5 has a higher ratio value than composite C5 (Figure 2). Analysis also indicates the highest proportion of the amorphous and crystalline phases in composite A1 (without glycerol), which exhibits the highest ratio value, whereas composite A3 (containing 2 g of glycerol) shows the lowest (Figure 3). This clearly indicates the influence of glycerol in weakening both the crystalline and amorphous phases. According to the literature, the addition of glycerol reduces crystallinity [24], and the present results further demonstrate that MMT exerts a similar effect—higher MMT content corresponds to lower ratio values in both analyzed regions (Figure 2). These findings suggest that both glycerol and MMT disrupt the spatial organization of the composite. The structure becomes ordered without crystal growth or an increase in amorphous content, which is why a simultaneous decrease in both the amorphousness and crystallinity of the material is observed. This may indicate the formation of structures that do not correspond to either a classical crystalline or amorphous phase. Moreover, MMT may stiffen the composite matrix, leading to smaller or less ordered crystals.

In summary:

- Glycerol reduces the degree of cross-linking by competing for reactive hydroxyl and carboxyl groups through hydrogen bonding.
- Glycerol promotes polymer chain cleavage, leading to shorter chains and distinct structural organization compared with glycerol-free composites.
- The protonation state of carboxyl groups depends on composition: in glycerol-containing composites, esterification-driven consumption of –OH groups promotes deprotonation (–COO^−^), whereas in A1 (no MMT) and B7 (1% *m*/*v* MMT concentration) the protonated form (–COOH) is predominant. This reflects the dual role of MMT as a site for ion exchange and a potential “proton trap”.
- MMT content enhances molecular interactions and increases structural density by providing exchangeable surfaces and promotes hydrogen bonding through accessible –OH groups; however, this effect is partly counteracted by glycerol.
- MMT stiffens the polymer matrix and restricts chain mobility, while glycerol interacts with MMT layers through hydrogen bonding, reducing this stiffening effect (softens the structure) and stabilizing the spatial arrangement.
- Both glycerol and MMT simultaneously reduce crystallinity and amorphous content, disrupting conventional structural organization.
- The preceding point suggests the formation of intermediate, interfacial structures that do not correspond to purely crystalline or amorphous phases.
- Additional stiffening by MMT results in smaller or less ordered crystals.
- Consequently, glycerol and MMT partially neutralize each other’s adverse effects, leading to composites with balanced structural properties.
- Spectral similarities between composites C5 and C7 indicate that glycerol contributes to a more coherent chemical structure, in contrast to the greater variation observed in glycerol-free composites.

### 2.3. Scanning Electron Microscopy (SEM)

**Figure 5:**
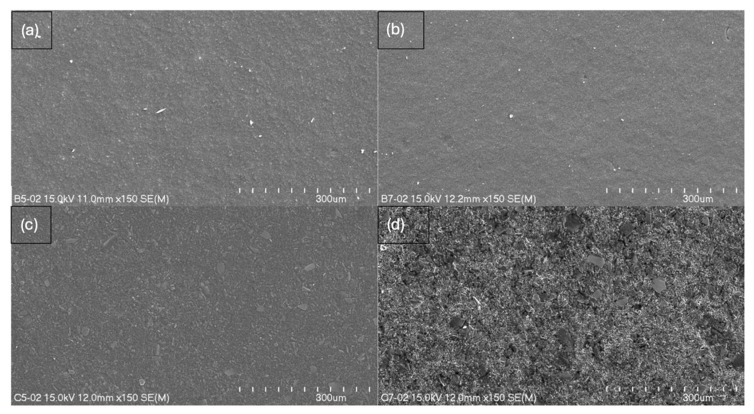
Scanning electron micrographs at 150× magnification (length scale 300 μm) of (**a**) B5, (**b**) B7, (**c**) C5, and (**d**) C7 composites.

**Figure 6:**
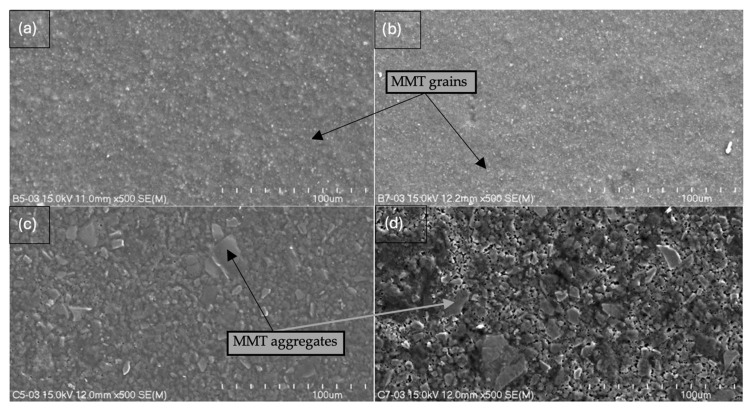
Scanning electron micrographs at 500× magnification (length scale 100 μm) of (**a**) B5, (**b**) B7, (**c**) C5, and (**d**) C7 composites.

**Figure 7:**
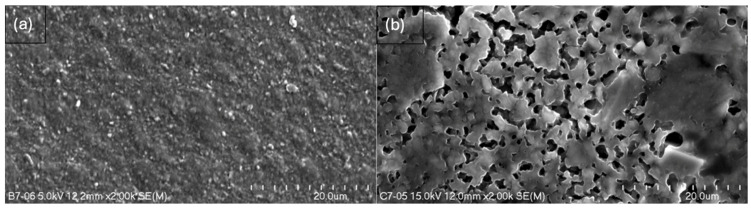
Scanning electron micrographs at 2000× magnification (length scale 20 μm) of (**a**) B7 and (**b**) C7.

**Figure 8:**
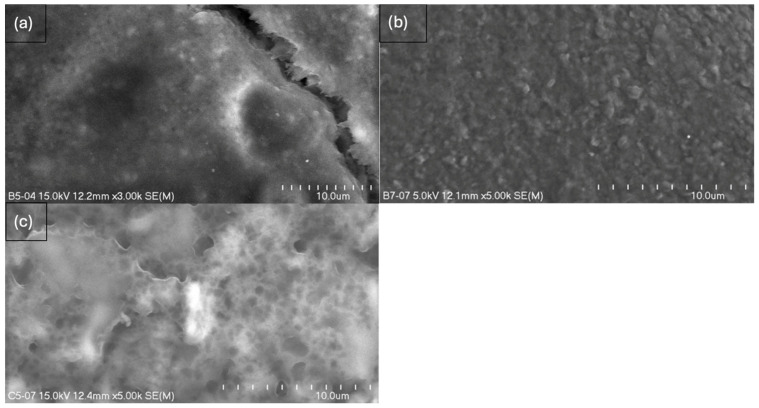
Scanning electron micrographs at 3000× magnification (length scale 10 μm) of composites (**a**) B5 and (**b**) B7 and at 5000× of composite (**c**) C5.

**Figure 9:**
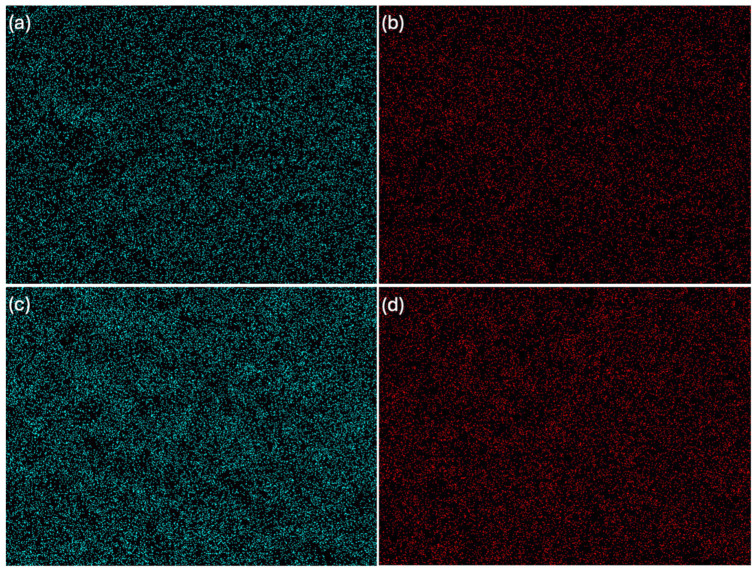
EDS elemental distribution maps: (**a**) chemical mapping of Si in B5, (**b**) chemical mapping of Al in B5, (**c**) chemical mapping of Si in C5, (**d**) chemical mapping of Al in C5.

**Figure 10:**
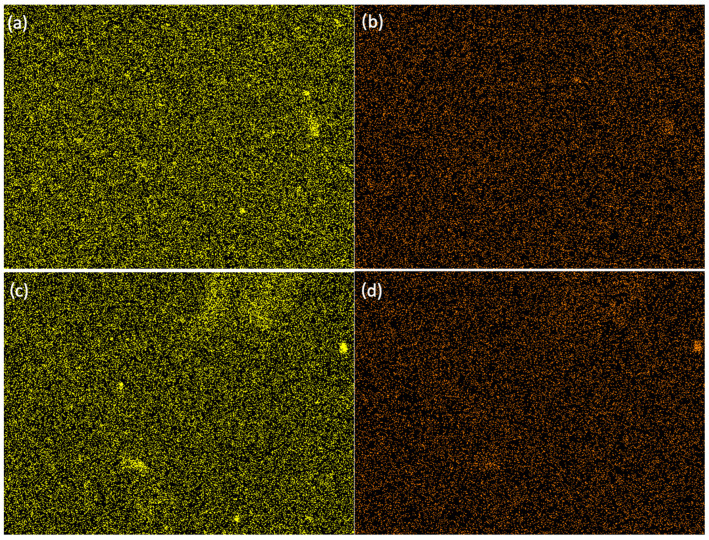
EDS elemental distribution maps: (**a**) chemical mapping of Si in B7, (**b**) chemical mapping of Al in B7, (**c**) chemical mapping of Si in C7, (**d**) chemical mapping of Al in C7.

The study resulted in images of B5, B7, C5, and C7 composites at magnifications of 150× (Figure 5), 500× (Figure 6), and 2000× (Figure 7) and/or 3000× and/or 5000× (Figure 8) at an accelerating voltage of 15 kV (the exception was B7 composite due to faster rate of material degradation under the beam; 5 kV was used at 2000× and 5000× magnifications). Composites B5 and B7 exhibited greater homogeneity compared to composites C5 and C7 (Figure 5 and Figure 6). Furthermore, no pores or larger granules/agglomerates were observed on the surfaces of composites B5 and B7. In contrast, the surfaces of composites C5 and C7 were significantly less homogeneous (Figure 5 and Figure 6), with the presence of larger grain clusters (Figure 6a,b versus Figure 6c,d). B5 appears to be smoother than B7 (Figure 8a,b), and B5 is the smoothest among the composites. C5 seems to be smoother than C7 (Figure 5c,d and Figure 6c,d). C5 and C7 exhibit a noticeably different spatial organization compared to B5 and B7 (Figure 6a,b vs. Figure 6c,d and Figure 7a,b), and the only difference between them is the presence (C-composites) or absence (B-composites) of glycerin. It was assumed that glycerol plays a key role in the spatial organization of the composite. B5 is smoother than B7, and C5 is smoother than C7 because the higher concentration of MMT results in a more granular and rougher structure. Composite C7 appears more promising in terms of drug incorporation. Scans show a good level of montmorillonite dispersion. To assess the homogeneous distribution of montmorillonite grains, EDS elemental distribution maps were acquired for the constituent chemical elements of the mineral, including Si and Al (further details can be found in the Supplementary Materials, Figure S2) in samples B5, C5, B7, and C7. Elemental EDS mapping was acquired over 30 min at an accelerating voltage of 15 keV, a beam current of 10 µA, and a working distance (WD) of 12 mm. As illustrated in Figure 9 and Figure 10, the chemical mapping of Si and Al indicates the uniform spatial distribution of montmorillonite grains in the B5, B7, C5, and C7 samples.

In summary:

- Composites without glycerol (B5, B7) exhibited greater homogeneity and an absence of cavities, pores, or larger spaces, while the addition of glycerol altered the spatial configuration of the composite, leading to the formation of pores or even larger spaces, as observed in C7.
- The results confirm the synergistic effect of glycerol and MMT; nevertheless, the dominant role of glycerol in promoting porosity formation is evident.
- The more homogeneous surface of composite B7 creates the possibility of the adhesion of active molecules on the surface—higher MMT content enhances the potential for hydrogen bond formation compared to B5, which is consistent with FTIR evidence of greater molecular interactions.
- The structure of C7 suggests the possibility of stronger particle binding and potential molecule entrapment within the matrix, which is especially relevant in the context of modified drug release.

### 2.4. Atomic Force Microscopy (AFM)

**Figure 11:**
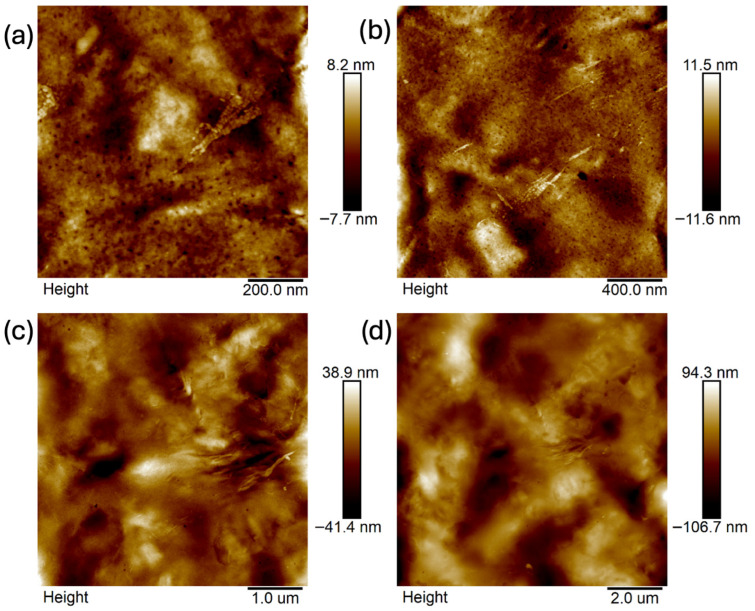
AFM: Topography images of composite B5: (**a**) 1 μm, (**b**) 2 μm, (**c**) 5 μm, (**d**) 10 μm.

**Figure 12:**
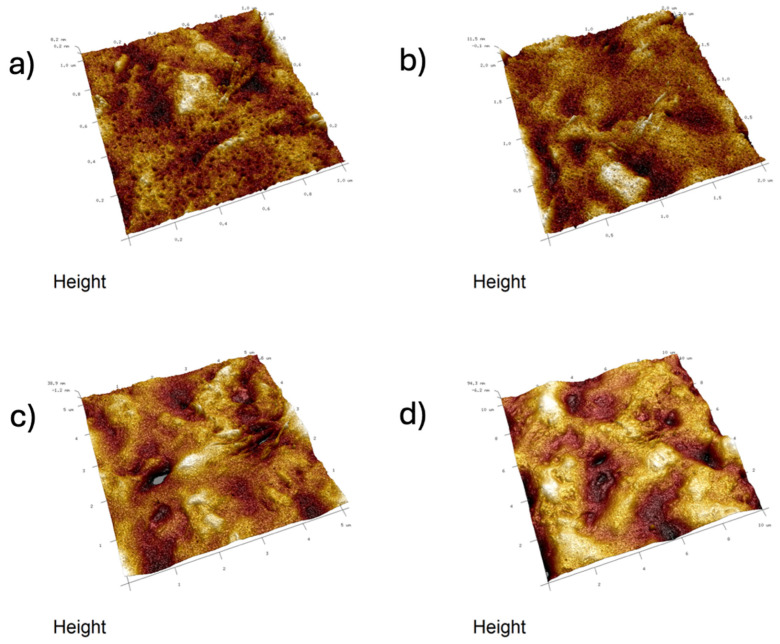
AFM: Three-dimensional images of composite B5: (**a**) 1 μm, (**b**) 2 μm, (**c**) 5 μm, (**d**) 10 μm.

**Figure 13:**
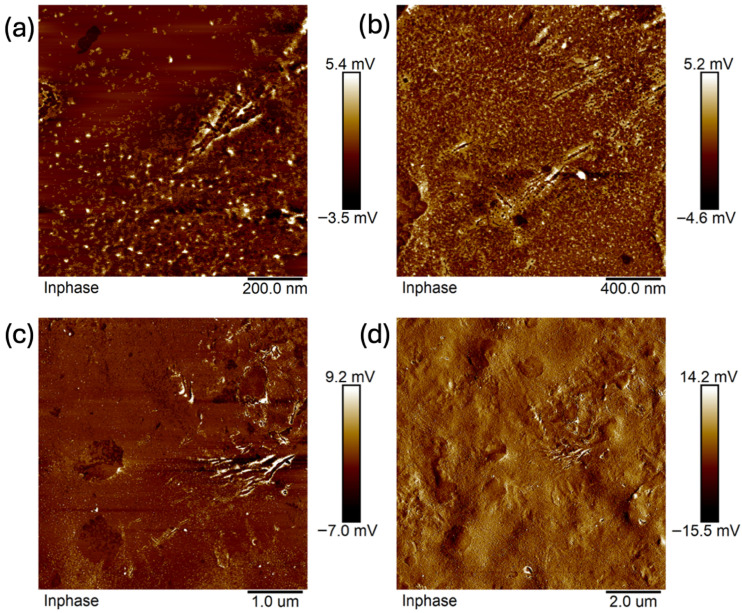
AFM: Phase images of composite B5: (**a**) 1 μm, (**b**) 2 μm, (**c**) 5 μm, (**d**) 10 μm.

**Figure 14:**
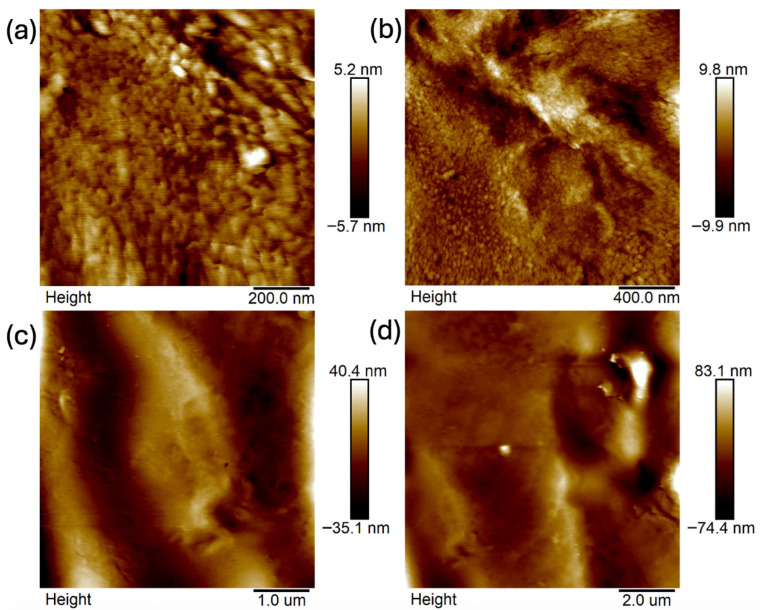
AFM: Topography images of composite B7: (**a**) 1 μm, (**b**) 2 μm, (**c**) 5 μm, (**d**) 10 μm.

**Figure 15:**
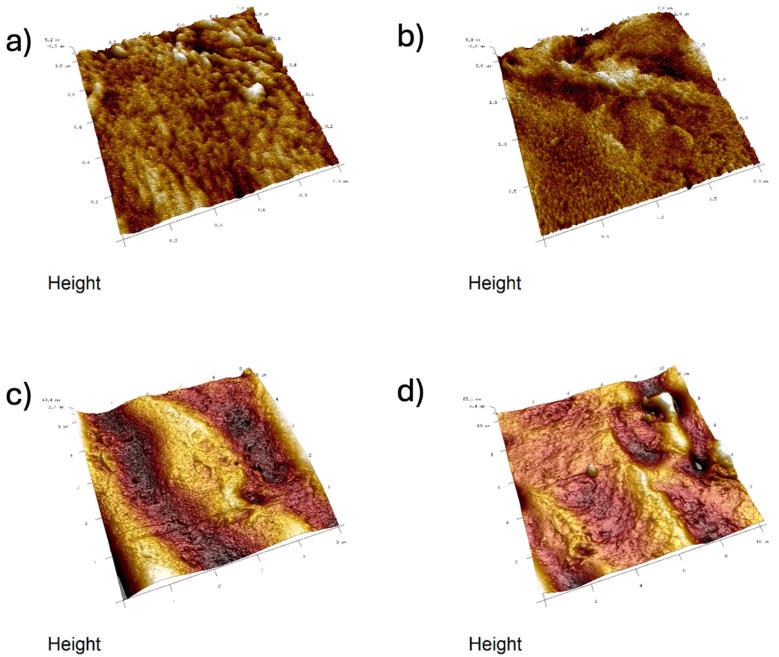
AFM: Three-dimensional images of composite B7: (**a**) 1 μm, (**b**) 2 μm, (**c**) 5 μm, (**d**) 10 μm.

**Figure 16:**
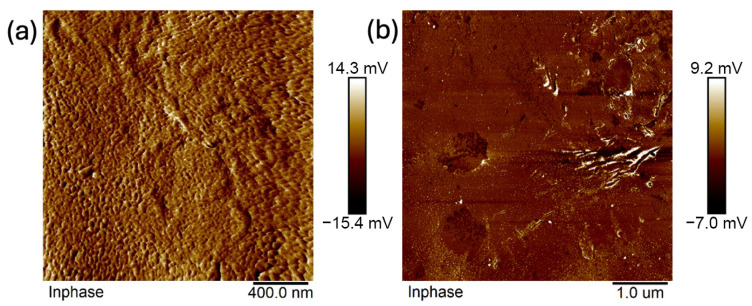
AFM: Phase images of composite B7: (**a**) 2 μm, (**b**) 5 μm.

**Figure 17:**
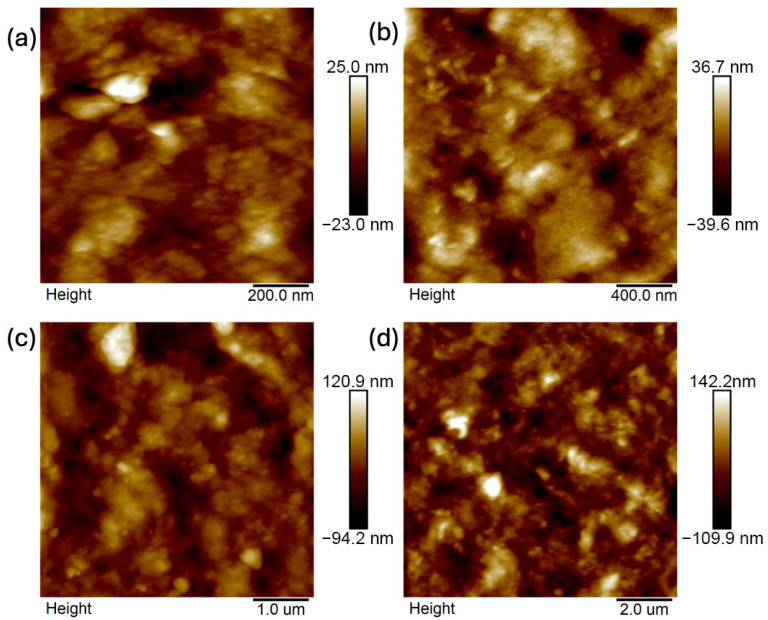
AFM: Topography images of composite C7: (**a**) 1 μm, (**b**) 2 μm, (**c**) 5 μm, (**d**) 10 μm.

**Figure 18:**
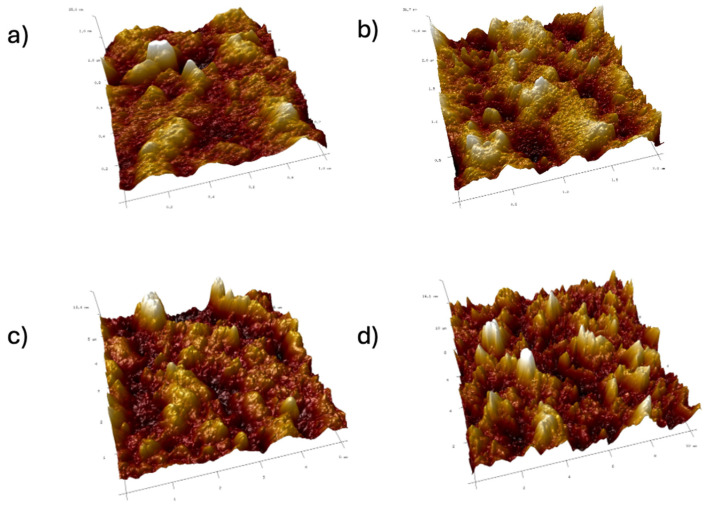
AFM: Three-dimensional images of composite C7: (**a**) 1 μm, (**b**) 2 μm, (**c**) 5 μm, (**d**) 10 μm.

**Figure 19:**
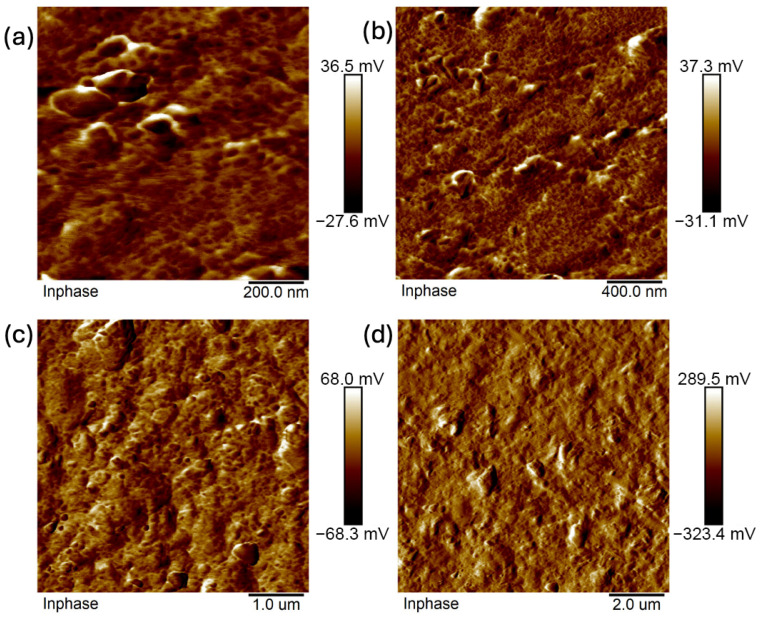
AFM: Phase images of composite C7: (**a**) 1 μm, (**b**) 2 μm, (**c**) 5 μm, (**d**) 10 μm.

Images were taken at 1 × 1 μm, 2 × 2 μm, 5 × 5 μm, and 10 × 10 μm (Figure 11, Figure 12, Figure 13, Figure 14, Figure 15, Figure 16, Figure 17, Figure 18 and Figure 19), and the roughness was determined using NanoScope Analysis 1.9 software. The parameters R_a_ (Average Roughness), R_q_ (Root Mean Square Roughness), and R_max_ (Maximum Roughness Depth) were collected and are presented in Table 2 (more details in Table S1). The data were taken from about five micrographs of each sample surface and then presented as the average value. The received data show that the roughness of composites B5 and B7, for scans of the same size (differing only in montmorillonite concentration), is similar. For composites B7 and C7 (differing only in the glycerol content), the roughness increases by up to several times when considering all parameters: R_a_, R_q_, R_max_. This is also reflected in the topographic images—the surface of composite C7 (Figure 17) is more differentiated than that of composite B7 (Figure 14). Grains with a diameter of approximately 80–250 nm are visible on its surface, forming cauliflower-shaped clusters with significant spaces between them. In some areas, single, isolated grains/conglomerates with a diameter of approximately 200–450 nm are also present. In addition, the surface is strongly wavy. All of these features contribute to the increase in roughness parameters. The surface of composite B7 (Figure 14) is more uniform, covered with grains of a similar diameter, approximately 50–100 nm. Its roughness is mainly due to the surface undulations while maintaining its continuity, resulting in lower roughness parameters compared to composite C7. The same described trend is possible to be observed in images of different sizes, although the R_a_, R_q_, and R_max_ parameters vary for each scan. The increase in their values along with the increasing scan area is obvious and easy to predict but still points to the same relationship between changes in the roughness of particulate samples. The inphase signal image shows the distribution of the mechanical-viscosity properties on the surface of the sample. Its variations result, among other things, from changes in chemical composition or charge [27]. Therefore, the phase images confirm the greater surface homogeneity of composite B7 (Figure 16) compared to composite C7 (Figure 19). While MMT aggregation is generally considered a disadvantage, the authors view it as a potential opportunity for the incorporation of specific particles [28]. As noted by the authors of another study [18], aggregation occurs with increased MMT content in the composite. In this study, however, glycerol played a key role. It caused both a change in the spatial organization of the composite (visible on the SEM and AFM scans and also in more detail on the FTIR spectra) and an increase in the material’s surface roughness.

In summary:

- Analysis confirmed an increase in surface roughness, as well as an appearance of larger grains, pores, and spaces in the material after the addition of glycerol. These are positive phenomena both in terms of adhesion of the dressing to the wound and enabling drug incorporation.
- The occurrence of MMT aggregates in the C7 matrix (with higher MMT content and glycerol) was also indicated. While MMT aggregation is generally considered a disadvantage, in this case, it may represent an opportunity for the targeted incorporation or immobilization of active molecules within the matrix.

## 3. Materials and Methods

### 3.1. Materials

Sodium carboxymethyl cellulose powder (CMC, DS = 0.5–0.7, viscosity of a 2% solution according to Höppler at 20 °C [mPas] ≤ 3000) was purchased from CMC S.A., Radom, Poland, Poly(vinyl alcohol) (PVA, M_w_ ~61,000, viscosity = 9.0–11.0 mPas, 98.0–98.8 mol% hydrolysis) was obtained from Kuraray, Frankfurt am Main, Germany, glycerin and citric acid (CA) were purchased from Stanlab, Lublin, Poland. Montmorillonite (MMT) was obtained from bentonite (Bentonit SN, Zębiec, Poland) by the purification method used by Kelar et al. [29], as briefly described in Preparation of Composite Materials.

According to the previously mentioned validated method, the purified MMT fraction obtained from bentonite exhibits an average particle size in the range of 12–22 µm and a cation exchange capacity (CEC) of approximately 100–110 mmol/100 g. The yield of the purification process was about 15%.

### 3.2. Preparation Method

Raw bentonite was put into a measuring cylinder, to which Milli-Q water was added. Then, the suspension was shaken and left for 72 h. After 3 days, the aqueous dispersion of bentonite was gently decanted and centrifugated for 25 min at 5000 rpm. The sediment deposited at the bottom of the tube after centrifugation was collected and dried to a constant mass in the oven at 80 °C. The montmorillonite fraction obtained in this way was ground using an agate mortar and pestle [29].

**Figure 20:**
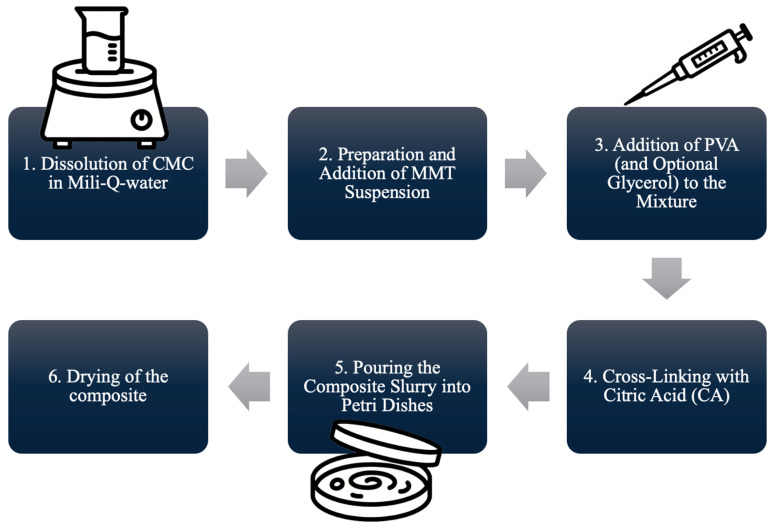
Composite synthesis scheme.

A 3.5 g amount of MMT was put into the beaker, into which 50 mL of Milli-Q water was poured, and stirred for 60 min at 90 °C. Into another beaker, 0.25 g of CMC was put, with 10 mL of Milli-Q water added, and stirred for 30 min at room temperature. An adequate amount of MMT suspension was added to the dissolved CMC and stirred for 30 min at room temperature. Then, 1 g of PVA was dissolved in 10 mL of Milli-Q water at 90 °C, and glycerin was added (or not, according to Table 3). The PVA mixture was added to CMC-MMT and stirred for 30 min. Then, the mixture was heated to 80 °C, and 10 mL of 0.3% CA was added as a crosslinker. The obtained slurry was stirred for 30 min, then cooled down, poured into a Petri dish, and dried in the oven for 48 h at 40 °C (Figure 20). Composites A1, A2, and A3 were used as reference composites, and composites B5, B7, C5, and C7 were examined materials.

### 3.3. Instrumentation

Attenuated total reflectance-Fourier transform infrared spectroscopy (ATR-FTIR) was performed on a Nexus FTIR Spectrometer Thermo Nicolet with the Golden Gate ATR attachment (Software Omnic 5.2a, ThermoNicolet Inc., Madison, WI, USA).

Scanning electron microscopy (SEM)—Surface morphologies of the synthesized hydrogel matrices were investigated using a HITACHI S-4700 microscope (Hitachi High-Tech Corporation, Tokyo, Japan) with a NORAN Vantage EDS microanalysis system (NORAN Instruments, Middleton, WI, USA). The samples were sputtered with carbon.

Atomic force microscopy (AFM)—The analysis was performed by means of a Bruker MULTIMODE 8 microscope (group of Scanning Probe Microscopies (SPM)) (Bruker Corporation, Billerica, MA, USA), applying AS-130 scanner (“J”) and SNL-10 probes. All measurements were performed in semi-contact mode: Tapping and Peak Force Tapping.

## 4. Conclusions

Composites based on PVA and CMC, modified with glycerol and MMT, were designed and synthesized. The synthesis method was novel in terms of both duration (quick synthesis) and the sequence of composite preparation. The materials obtained using this method exhibited appropriate mechanical (in terms of brittleness and ductility) properties. SEM, FTIR, and AFM analyses were performed to confirm their expected properties. In summary, analysis confirmed the successful preparation of composites whose properties can be tuned through the combined use of glycerol and montmorillonite (MMT). Both act independently and cooperatively, enabling the design of cross-linking efficiency, protonation state, and structural organization. Their combined action generates hybrid structures that transcend classical crystalline–amorphous classification, offering new design strategies for functional polymer composites with tunable properties. When considered alongside FTIR findings, SEM results reinforce the conclusion that glycerol is the principal factor responsible for disrupting compact molecular packing and promoting alternative structural arrangements, while MMT modulates these effects by stabilizing or compensating them. AFM observations complement SEM findings of enhanced porosity and further support the FTIR evidence of disrupted molecular organization induced by glycerol. Together, the two additives generate hybrid architectures characterized by both interfacial porosity and altered molecular organization. Future studies involving XRD, DSC, and rheology, together with determination of the swelling degree (SD) and gel fraction (GF), will provide a more comprehensive characterization of the composites. These analyses will allow evaluation the absorbency of the prospective dressing material and its thermal and rheological properties, and will also provide further insights into the composite structure and help determine whether the synthesized systems are suitable as multifunctional wound dressings and drug delivery carriers.

## Figures and Tables

**Table 1:** FTIR peak assignments and their corresponding functional groups observed in composites.

Wavenumber [cm^−1^]	Characteristic Bond and Movement	Ref.
3266	O–H stretching vibrations	[21,22]
2939, 2908	stretching vibrations of the –CH_2_	[11,18,21,22,24]
2850	stretching vibrations of the –CH_3_	[11]
1710	stretching vibrations of the C=O bond in ester groups that were formed by CA	[21,22,23]
1641, 1594	asymmetric vibrations of the COO^−^ group	[11,23]
1415, 1322	symmetric vibrations of the COO^−^ group	[11,22,23]
1238	stretching vibrations related to ester bonds	[23]
1142, 1083, 1034	stretching vibrations of the C–O bond	[11,18,23,24]

**Table 2:** The parameters R_a_ (Average Roughness), R_q_ (Root Mean Square Roughness) and R_max_ (Maximum Roughness Depth) of the samples based on scans of different dimensions.

Composite	1 × 1 μm			2 × 2 μm			5 × 5 μm			10 × 10 μm		
	R_a_ [nm]	R_q_ [nm]	R_max_ [nm]	R_a_ [nm]	R_q_ [nm]	R_max_ [nm]	R_a_ [nm]	R_q_ [nm]	R_max_ [nm]	R_a_ [nm]	R_q_ [nm]	R_max_ [nm]
**B5**	1.798 (0.196)	2.366 (0.278)	21.68 (2.167)	2.47 (0.089)	3.247 (0.127)	40.97 (2.702)	8.18 (0.078)	10.67 (0.058)	92.87 (2.04)	22.43 (0.058)	28.2 (0.01)	228 (0.0)
**B7**	0.979 (0.091)	1.297 (0.121)	15.6 (2.629)	3.417 (1.606)	4.27 (1.806)	33.15 (8.458)	14.636 (5.670)	17.775 (6.458)	118.35 (14.911)	16.95 (3.748)	23.45 (4.738)	263.5 (47.376)
**C7**	5.3 (1.788)	6.797 (2.231)	51.6 (8.404)	9.077 (0.985)	11.567 (1.172)	88.8 (14.031)	25.4 (3.559)	32.7 (4.423)	235 (36.116)	27.05 (1.345)	36.0 (2.121)	389 (16.971)

**Table 3:** Hydrogel formulae and MMT concentrations in composites.

Composite	CMC [g]	PVA [g]	0.3% CA [mL]	Glycerin [g]	MMT Concentration [*m*/*v* %]
**A1**	0.25	1.00	10.00	0.00	0.00
**A2**	0.25	1.00	10.00	1.00	0.00
**A3**	0.25	1.00	10.00	2.00	0.00
**B5**	0.25	1.00	10.00	0.00	0.25
**B7**	0.25	1.00	10.00	0.00	1.00
**C5**	0.25	1.00	10.00	1.00	0.25
**C7**	0.25	1.00	10.00	1.00	1.00

## Data Availability

The original contributions presented in this study are included in the article/Supplementary Material. Further inquiries can be directed to the corresponding author.

## Supplementary Materials

The supporting information can be downloaded at: https://www.mdpi.com/article/10.3390/ijms262311445/s1.
