# Supplementary material for: Physicochemical Characterization and Structural Tuning of PVA/CMC Composites Modified with Montmorillonite and Glycerol: Toward Sustainable Wound Dressings
Source: Int J Mol Sci. 2025 Nov 26;26(23):11445. doi: 10.3390/ijms262311445 (PMC12692337; doi:10.3390/ijms262311445)
Supplement: Supplementary file 1 [file ijms-26-11445-s001.zip › ijms-3983664-supplementary.pdf]

Physicochemical Characterization and Structural Tuning of PVA/CMC Composites Modified with Montmorillonite and Glycerol: Toward Sustainable Wound Dressings

Aleksandra Niedźwiecka[1], Irena Brunarska[2], Anna Adamczyk[3], Przemysław Talik[1,4*]

[1] University of Opole, Faculty of Chemistry and Pharmacy, Oleska 48, 45-052 Opole, Poland; aleksandra.niedzwiecka@uni.opole.pl,

[2] Jagiellonian University, Scanning Electron Microscopy and Microanalysis Laboratory

Institute of Geological Sciences, Gronostajowa 3a, 30-387 Krakow, Poland; irena.brunarska@uj.edu.pl

[3] AGH University of Krakow, Faculty of Materials Science and Ceramics, Krakow, Poland; aadamcz@agh.edu.pl

[4] Jagiellonian University Medical College, Faculty of Pharmacy, 9 Medyczna St., Krakow, 30-688, Poland

* Correspondence: przemyslaw.talik@uni.opole.pl;

Table S1. Extended data of the parameters Ra (Average Roughness), Rq (Root Mean Square Roughness) and Rmax (Maximum Roughness Depth) of the samples based on scans of different dimensions ……………………………………………………………………………….....2

Figure S1. Elemental data from SEM (composites B5,B7,C5,C7) ………………………………..3-14

Table S1. Extended data of the parameters Ra (Average Roughness), Rq (Root Mean Square Roughness) and Rmax (Maximum Roughness Depth) of the samples based on scans of different dimensions.

composite	1mm			2mm			5mm			10mm		
	R_a [nm]	R_q [nm]	R_{max} [nm]	R_a [nm]	R_q [nm]	R_{max} [nm]	R_a [nm]	R_q [nm]	R_{max} [nm]	R_a [nm]	R_q [nm]	R_{max} [nm]
**B5**	1.62	2.11	21.7	2.37	3.11	37.9	8.09	10.6	95.1	22.4	28.2	228
	1.58	2.05	20.4	2.5	3.27	42	8.22	10.7	91.1	22.4	28.1	228
	1.87	2.48	20.08	2.54	3.36	43	8.23	10.7	92.4	22.5	28.3	228
	1.87	2.48	20.8	-	-	-	-	-	-	-	-	-
	2.05	2.71	25.4	-	-	-	-	-	-	-	-	-
**Average**	1.80	2.37	21.68	2.47	3.25	40.97	8.18	10.67	92.87	22.43	28.2	228
st dev	0.20	0.28	2.17	0.09	0.13	2.70	0.08	0.06	2.04	0.06	0.1	0
**B7**	1.08	1.41	13.7	1.89	2.52	25.3	10.5	12.5	96.4	14.3	20.1	230
	0.957	1.31	18.6	3.77	4.66	41.1	20.1	23.9	129	19.6	26.8	297
	0.901	1.17	14.5	5.52	6.63	39.8	18.9	22.8	126	-	-	-
	-	-	-	2.49	3.27	26.4	9.04	11.9	122	-	-	-
**Average**	0.98	1.30	15.6	3.42	4.27	33.15	14.64	17.775	118.35	16.95	23.45	263.5
st dev	0.09	0.12	2.63	1.61	1.81	8.46	5.67	6.46	14.92	3.75	4.74	47.38
**C7**	4.61	6.11	52.7	8.36	10.7	80.5	21.3	27.6	194	26.1	34.5	377
	7.33	9.29	59.4	8.67	11.1	80.9	27.2	35	258	28	37.5	401
	3.96	4.99	42.7	10.2	12.9	105	27.7	35.5	255	-	-	-
**Average**	5.3	6.79	51.6	9.08	11.57	88.8	25.4	32.7	235.67	27.05	36	389
st dev	1.78	2.23	8.40	0.98	1.17	14.03	3.56	4.43	36.12	1.34	2.12	16.97

Figure S1. Elemental data from SEM (composites B5,B7,C5,C7).

B5-03(1)

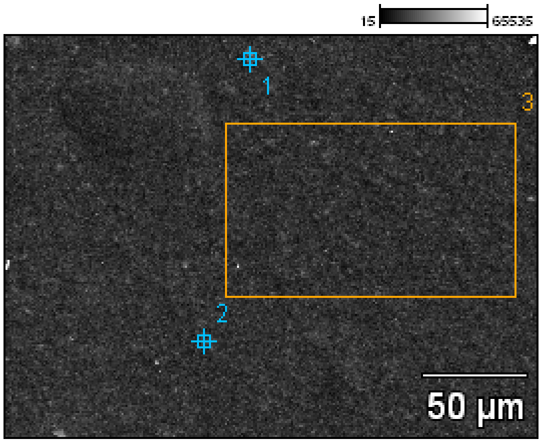

Image Name: B5-03(1)

Accelerating Voltage: 15.0 kV

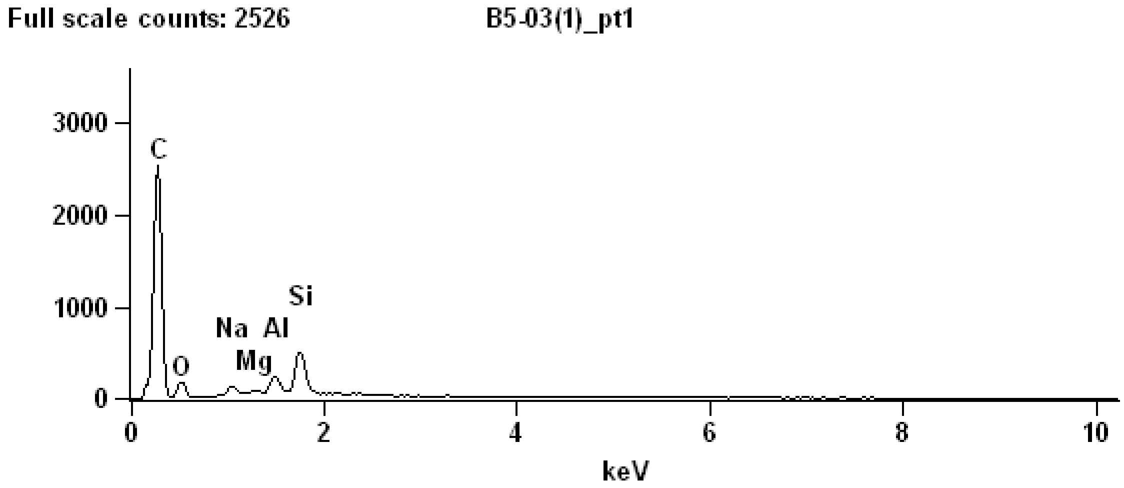

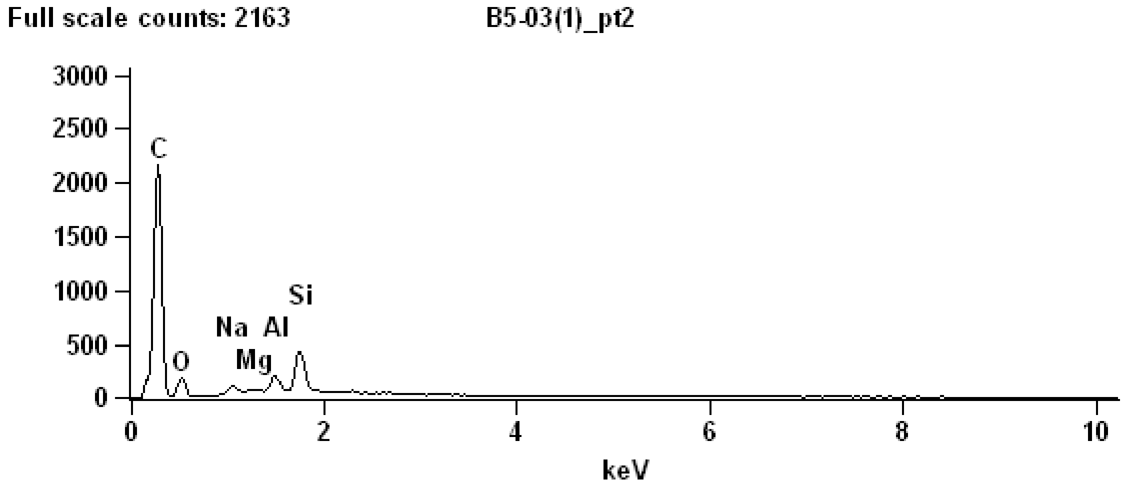

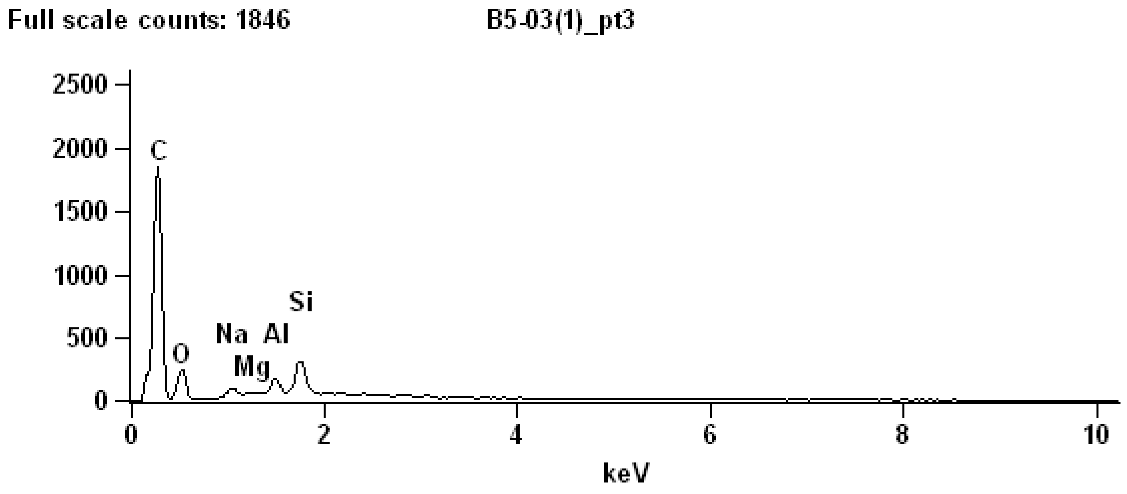

Full scale counts: 1846
B5-03(1)_pt3
2500
2000
1500
1000
500
0
C
O
Na
Mg
Al
Si
0
2
4
6
8
10
keV

Net Counts

	O-K	Na-K	Mg-K	Al-K	Si-K
B5-03(1)_pt1	1199	856	174	1608	4922
B5-03(1)_pt2	1282	604	181	1277	3996
B5-03(1)_pt3	1869	620	137	901	2735

Net Counts Error (+/- 1 Sigma)

	O-K	Na-K	Mg-K	Al-K	Si-K
B5-03(1)_pt1	+/-50	+/-56	+/-49	+/-93	+/-118
B5-03(1)_pt2	+/-50	+/-51	+/-46	+/-87	+/-110
B5-03(1)_pt3	+/-57	+/-49	+/-39	+/-47	+/-98

Weight %

	O-K	Na-K	Mg-K	Al-K	Si-K
B5-03(1)_pt1	44.13	9.10	1.47	10.95	34.36
B5-03(1)_pt2	49.59	7.57	1.74	9.88	31.21
B5-03(1)_pt3	60.61	8.62	1.45	7.46	21.87

Weight % Error (+/- 1 Sigma)

	O-K	Na-K	Mg-K	Al-K	Si-K
B5-03(1)_pt1	+/-1.84	+/-0.60	+/-0.41	+/-0.63	+/-0.82
B5-03(1)_pt2	+/-1.93	+/-0.64	+/-0.44	+/-0.67	+/-0.86
B5-03(1)_pt3	+/-1.85	+/-0.68	+/-0.41	+/-0.39	+/-0.78

Atom %

	O-K	Na-K	Mg-K	Al-K	Si-K
B5-03(1)_pt1	56.95	8.17	1.25	8.38	25.26
B5-03(1)_pt2	62.26	6.62	1.44	7.36	22.33
B5-03(1)_pt3	71.78	7.11	1.13	5.24	14.75

Atom % Error (+/- 1 Sigma)

	O-K	Na-K	Mg-K	Al-K	Si-K
B5-03(1)_pt1	+/-2.37	+/-0.53	+/-0.35	+/-0.48	+/-0.61
B5-03(1)_pt2	+/-2.43	+/-0.56	+/-0.37	+/-0.50	+/-0.61
B5-03(1)_pt3	+/-2.19	+/-0.56	+/-0.32	+/-0.27	+/-0.53

Formula

	O-K	Na-K	Mg-K	Al-K	Si-K
B5-03(1)_pt1	O	Na	Mg	Al	Si
B5-03(1)_pt2	O	Na	Mg	Al	Si
B5-03(1)_pt3	O	Na	Mg	Al	Si

Compound %

	O	Na	Mg	Al	Si
B5-03(1)_pt1	44.13	9.10	1.47	10.95	34.36
B5-03(1)_pt2	49.59	7.57	1.74	9.88	31.21
B5-03(1)_pt3	60.61	8.62	1.45	7.46	21.87

Cations

	O-K	Na-K	Mg-K	Al-K	Si-K
B5-03(1)_pt1	0.00	0.00	0.00	0.00	0.00
B5-03(1)_pt2	0.00	0.00	0.00	0.00	0.00
B5-03(1)_pt3	0.00	0.00	0.00	0.00	0.00

B7-04(1)

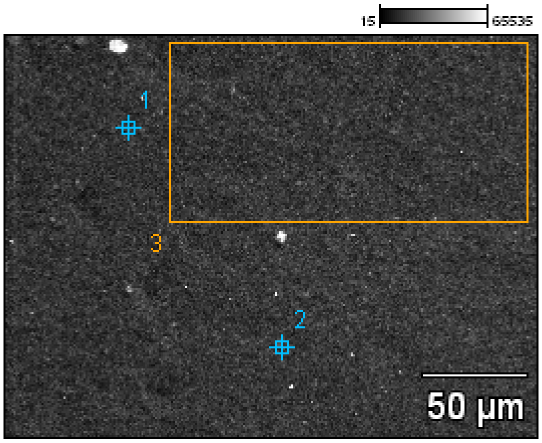

Image Name: B7-04(1)

Accelerating Voltage: 15.0 kV

Full scale counts: 1844 B7-04(1)_pt1

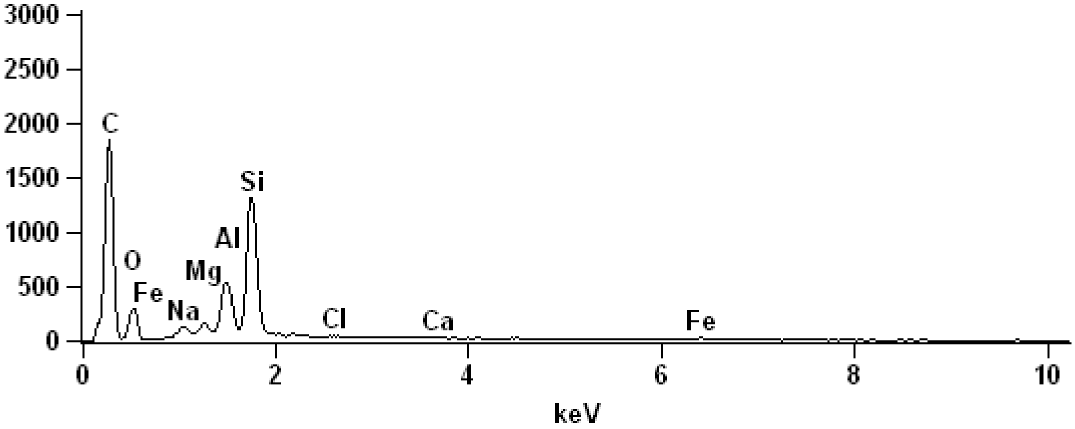

Full scale counts: 1324 B7-04(1)_pt2

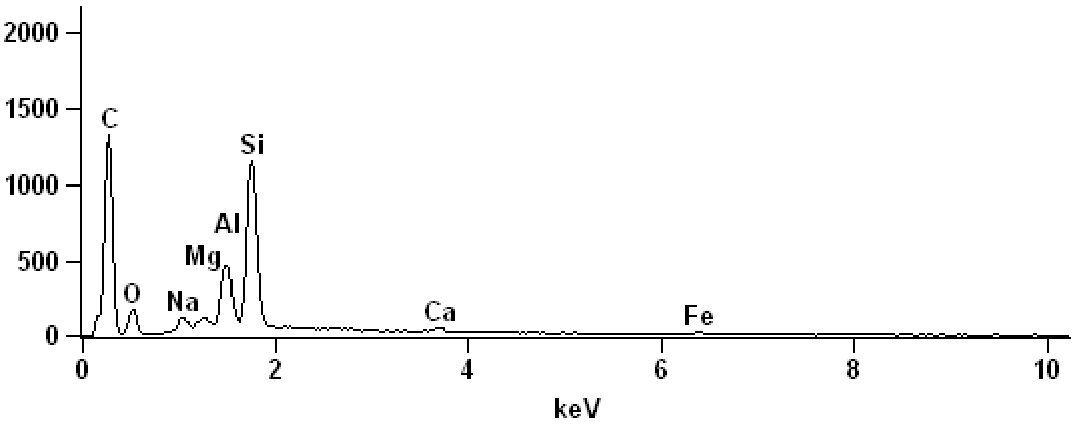

Full scale counts: 1699 B7-04(1)_pt3

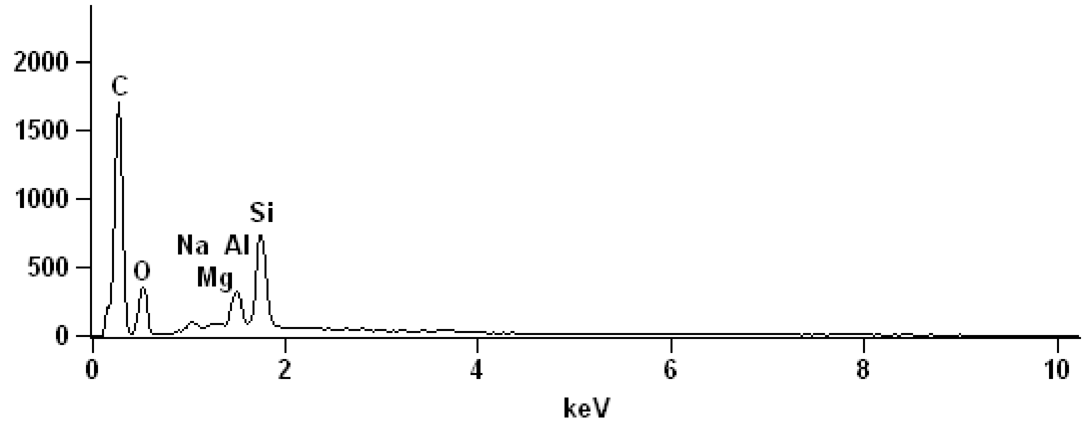

Net Counts

	O-K	Na-K	Mg-K	Al-K	Si-K	Cl-K	Ca-K	Fe-K
**B7-04(1)_pt1**	2197	807	627	4816	13433	156	269	395
**B7-04(1)_pt2**	1231	771	567	3987	11858		374	192
**B7-04(1)_pt3**	2584	602	310	2446	7036			

Net Counts Error (+/- 1 Sigma)

	O-K	Na-K	Mg-K	Al-K	Si-K	Cl-K	Ca-K	Fe-K
**B7-04(1)_pt1**	+/-69	+/-62	+/-60	+/-130	+/-175	+/-40	+/-38	+/-41
**B7-04(1)_pt2**	+/-53	+/-56	+/-81	+/-121	+/-165		+/-39	+/-36
**B7-04(1)_pt3**	+/-65	+/-53	+/-47	+/-104	+/-135			

Weight %

	O-K	Na-K	Mg-K	Al-K	Si-K	Cl-K	Ca-K	Fe-K
**B7-04(1)_pt1**	36.62	3.62	2.04	12.92	37.98	0.61	1.21	5.01
**B7-04(1)_pt2**	30.30	4.22	2.33	13.73	43.93		2.25	3.24
**B7-04(1)_pt3**	53.33	4.31	1.61	10.34	30.40			

Weight % Error (+/- 1 Sigma)

	O-K	Na-K	Mg-K	Al-K	Si-K	Cl-K	Ca-K	Fe-K
**B7-04(1)_pt1**	+/-1.15	+/-0.28	+/-0.20	+/-0.35	+/-0.49	+/-0.16	+/-0.17	+/-0.52
**B7-04(1)_pt2**	+/-1.30	+/-0.31	+/-0.33	+/-0.42	+/-0.61		+/-0.23	+/-0.61
**B7-04(1)_pt3**	+/-1.34	+/-0.38	+/-0.24	+/-0.44	+/-0.58			

Atom %

	O-K	Na-K	Mg-K	Al-K	Si-K	Cl-K	Ca-K	Fe-K
**B7-04(1)_pt1**	50.89	3.50	1.86	10.64	30.06	0.38	0.67	1.99
**B7-04(1)_pt2**	43.43	4.21	2.20	11.67	35.87		1.29	1.33
**B7-04(1)_pt3**	65.97	3.71	1.31	7.59	21.42			

Atom % Error (+/- 1 Sigma)

	O-K	Na-K	Mg-K	Al-K	Si-K	Cl-K	Ca-K	Fe-K
**B7-04(1)_pt1**	+/-1.60	+/-0.27	+/-0.18	+/-0.29	+/-0.39	+/-0.10	+/-0.09	+/-0.21
**B7-04(1)_pt2**	+/-1.87	+/-0.31	+/-0.31	+/-0.35	+/-0.50		+/-0.13	+/-0.25
**B7-04(1)_pt3**	+/-1.66	+/-0.33	+/-0.20	+/-0.32	+/-0.41			

Formula

	O-K	Na-K	Mg-K	Al-K	Si-K	Cl-K	Ca-K	Fe-K
**B7-04(1)_pt1**	O	Na	Mg	Al	Si	Cl	Ca	Fe
**B7-04(1)_pt2**	O	Na	Mg	Al	Si		Ca	Fe
**B7-04(1)_pt3**	O	Na	Mg	Al	Si			

Compound %

	O	Na	Mg	Al	Si	Cl	Ca	Fe
**B7-04(1)_pt1**	36.62	3.62	2.04	12.92	37.98	0.61	1.21	5.01
**B7-04(1)_pt2**	30.30	4.22	2.33	13.73	43.93		2.25	3.24
**B7-04(1)_pt3**	53.33	4.31	1.61	10.34	30.40			

Cations

	O-K	Na-K	Mg-K	Al-K	Si-K	Cl-K	Ca-K	Fe-K
**B7-04(1)_pt1**	0.00	0.00	0.00	0.00	0.00	0.00	0.00	0.00
**B7-04(1)_pt2**	0.00	0.00	0.00	0.00	0.00		0.00	0.00
**B7-04(1)_pt3**	0.00	0.00	0.00	0.00	0.00			

C5-05(1)

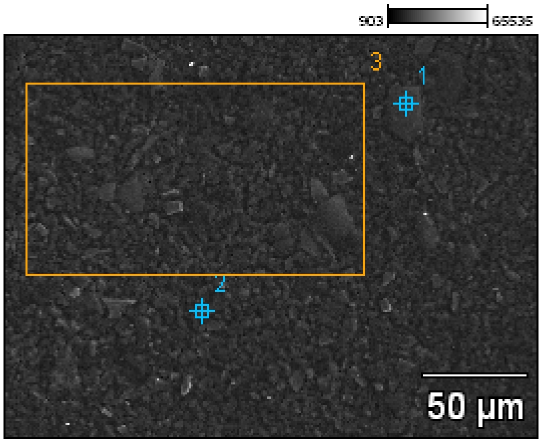

Image Name: C5-05(1)

Accelerating Voltage: 15.0 kV

Full scale counts: 2008 C5-05(1)_pt1

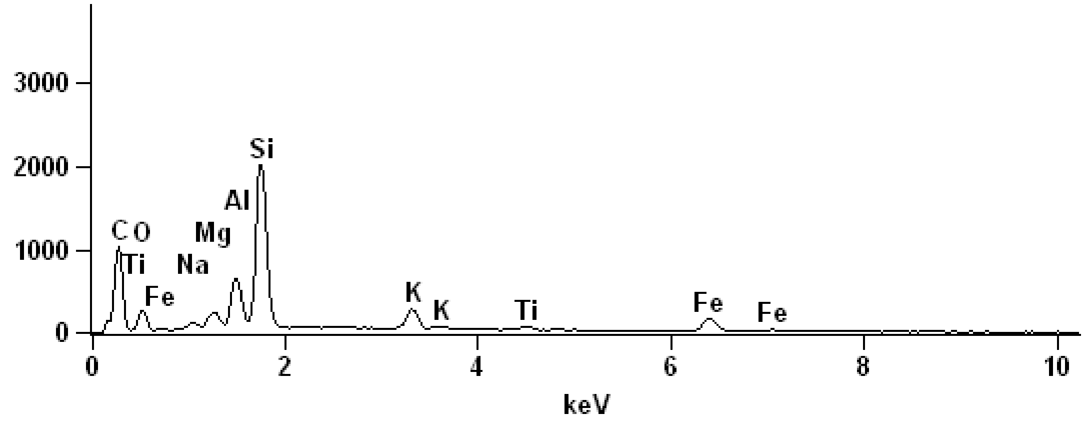

Full scale counts: 3629 C5-05(1)_pt2

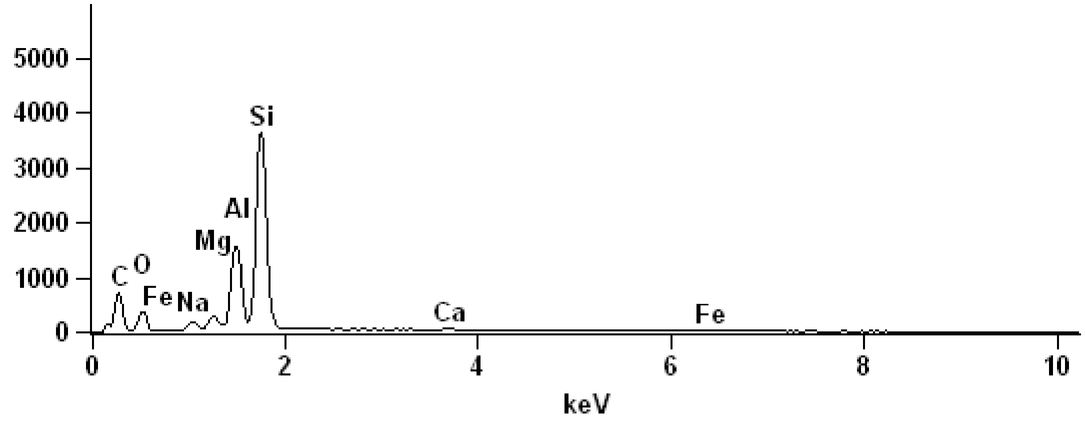

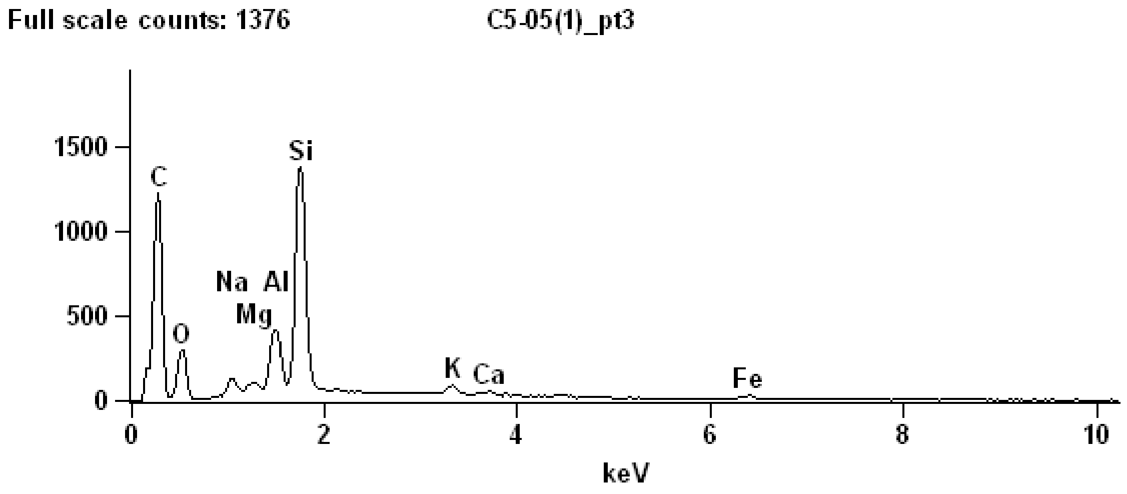
Full scale counts: 1376
C5-05(1)_pt3
1500
1000
500
0
C
O
Na
Mg
Al
Si
K
Ca
Fe
0
2
4
6
8
10
keV

Net Counts

	O-K	Na-K	Mg-K	Al-K	Si-K	K-K	Ca-K	Ti-K	Fe-K
C5-05(1)_pt1	2037	770	1875	5623	21879	3195		601	2328
C5-05(1)_pt2	2946	1356	1679	14872	39146		305		490
C5-05(1)_pt3	2317	889	470	3593	14513	612	222		324

Net Counts Error (+/- 1 Sigma)

	O-K	Na-K	Mg-K	Al-K	Si-K	K-K	Ca-K	Ti-K	Fe-K
C5-05(1)_pt1	+/-68	+/-60	+/-93	+/-145	+/-218	+/-120		+/-45	+/-118
C5-05(1)_pt2	+/-68	+/-69	+/-107	+/-214	+/-297		+/-45		+/-49
C5-05(1)_pt3	+/-62	+/-55	+/-54	+/-117	+/-176	+/-44	+/-40		+/-42

Weight %

	O-K	Na-K	Mg-K	Al-K	Si-K	K-K	Ca-K	Ti-K	Fe-K
C5-05(1)_pt1	23.49	2.26	3.77	9.25	35.37	7.34		2.17	16.36
C5-05(1)_pt2	25.71	2.40	2.20	16.66	49.56		0.63		2.83
C5-05(1)_pt3	38.93	3.95	1.52	9.43	38.72	2.47	0.98		4.01

Weight % Error (+/- 1 Sigma)

	O-K	Na-K	Mg-K	Al-K	Si-K	K-K	Ca-K	Ti-K	Fe-K
C5-05(1)_pt1	+/-0.78	+/-0.18	+/-0.19	+/-0.24	+/-0.35	+/-0.28		+/-0.16	+/-0.83
C5-05(1)_pt2	+/-0.59	+/-0.12	+/-0.14	+/-0.24	+/-0.38		+/-0.09		+/-0.28
C5-05(1)_pt3	+/-1.04	+/-0.24	+/-0.17	+/-0.31	+/-0.47	+/-0.18	+/-0.18		+/-0.52

Atom %

	O-K	Na-K	Mg-K	Al-K	Si-K	K-K	Ca-K	Ti-K	Fe-K
C5-05(1)_pt1	38.14	2.56	4.03	8.91	32.71	4.87		1.17	7.61
C5-05(1)_pt2	37.81	2.46	2.13	14.53	41.51		0.37		1.19
C5-05(1)_pt3	53.42	3.77	1.37	7.67	30.27	1.39	0.54		1.58

Atom % Error (+/- 1 Sigma)

	O-K	Na-K	Mg-K	Al-K	Si-K	K-K	Ca-K	Ti-K	Fe-K
C5-05(1)_pt1	+/-1.27	+/-0.20	+/-0.20	+/-0.23	+/-0.33	+/-0.18		+/-0.09	+/-0.39
C5-05(1)_pt2	+/-0.87	+/-0.13	+/-0.14	+/-0.21	+/-0.31		+/-0.05		+/-0.12
C5-05(1)_pt3	+/-1.43	+/-0.23	+/-0.16	+/-0.25	+/-0.37	+/-0.10	+/-0.10		+/-0.20

Formula

	O-K	Na-K	Mg-K	Al-K	Si-K	K-K	Ca-K	Ti-K	Fe-K
C5-05(1)_pt1	O	Na	Mg	Al	Si	K		Ti	Fe
C5-05(1)_pt2	O	Na	Mg	Al	Si		Ca		Fe
C5-05(1)_pt3	O	Na	Mg	Al	Si	K	Ca		Fe

Compound %

	O	Na	Mg	Al	Si	K	Ca	Ti	Fe
C5-05(1)_pt1	23.49	2.26	3.77	9.25	35.37	7.34		2.17	16.36
C5-05(1)_pt2	25.71	2.40	2.20	16.66	49.56		0.63		2.83
C5-05(1)_pt3	38.93	3.95	1.52	9.43	38.72	2.47	0.98		4.01

Cations

	O-K	Na-K	Mg-K	Al-K	Si-K	K-K	Ca-K	Ti-K	Fe-K
C5-05(1)_pt1	0.00	0.00	0.00	0.00	0.00	0.00		0.00	0.00
C5-05(1)_pt2	0.00	0.00	0.00	0.00	0.00		0.00		0.00
C5-05(1)_pt3	0.00	0.00	0.00	0.00	0.00	0.00	0.00		0.00

C7-03(1)

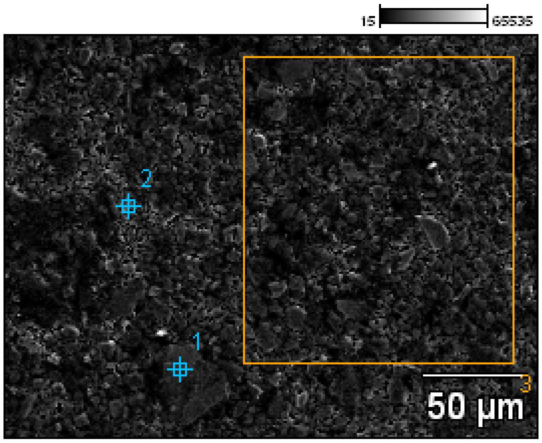

Image Name: C7-03(1)

Accelerating Voltage: 15.0 kV

Full scale counts: 2613 C7-03(1)_pt1

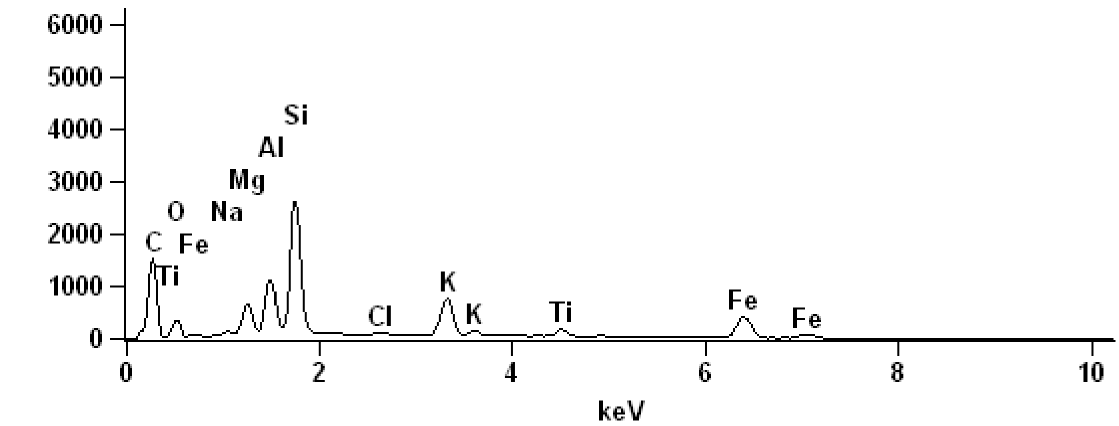

Full scale counts: 4544 C7-03(1)_pt2

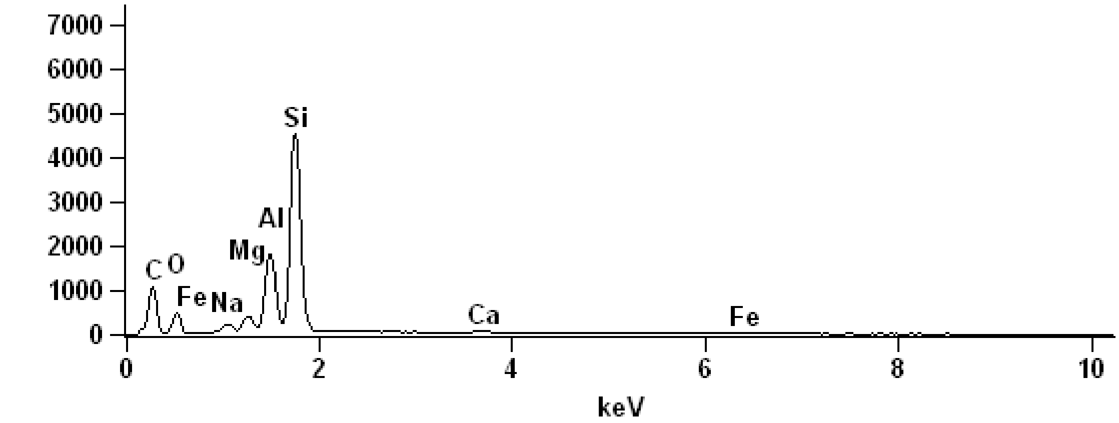

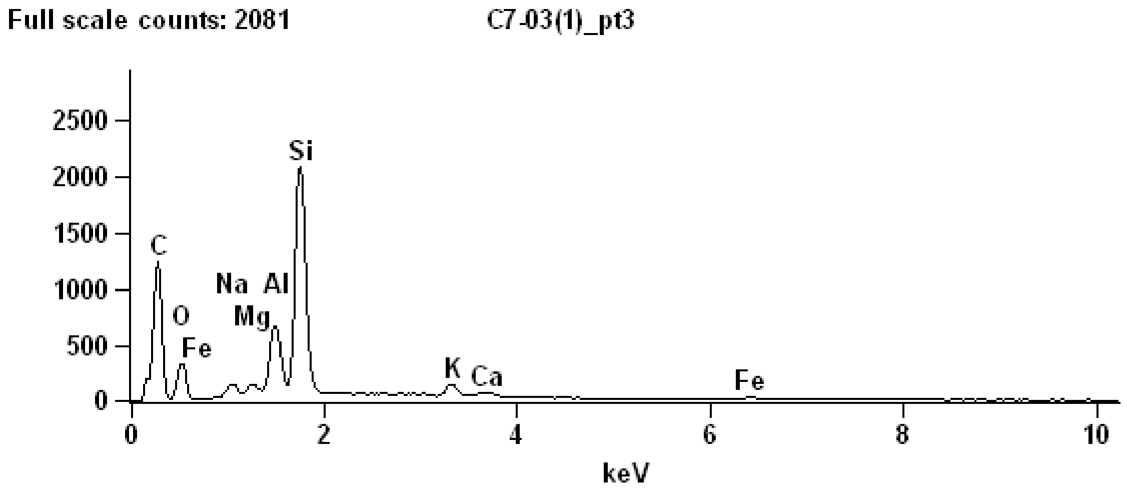

Full scale counts: 2081
C7-03(1)_pt3
2500
2000
1500
1000
500
0
Si
C
Na
Al
O
Mg
Fe
K
Ca
Fe
0
2
4
6
8
10
keV

Net Counts

	O-K	Na-K	Mg-K	Al-K	Si-K	Cl-K	K-K	Ca-K	Ti-K	Fe-K
**C7-03(1)_pt1**	2727	572	5201	9944	27824	508	9352		1841	6924
**C7-03(1)_pt2**	3814	1722	2881	16923	49262			1088		838
**C7-03(1)_pt3**	2608	1127	666	5883	22401		1249	453		454

Net Counts Error (+/- 1 Sigma)

	O-K	Na-K	Mg-K	Al-K	Si-K	Cl-K	K-K	Ca-K	Ti-K	Fe-K
**C7-03(1)_pt1**	+/-90	+/-54	+/-136	+/-202	+/-260	+/-62	+/-183		+/-113	+/-178
**C7-03(1)_pt2**	+/-88	+/-89	+/-130	+/-239	+/-342			+/-104		+/-58
**C7-03(1)_pt3**	+/-67	+/-62	+/-62	+/-144	+/-219		+/-53	+/-48		+/-85

Weight %

	O-K	Na-K	Mg-K	Al-K	Si-K	Cl-K	K-K	Ca-K	Ti-K	Fe-K
**C7-03(1)_pt1**	17.72	1.01	6.03	9.45	25.20	0.56	11.10		3.46	25.46
**C7-03(1)_pt2**	26.01	2.42	2.98	15.02	48.09			1.74		3.73
**C7-03(1)_pt3**	33.87	3.40	1.46	10.58	41.77		3.56	1.41		3.94

Weight % Error (+/- 1 Sigma)

	O-K	Na-K	Mg-K	Al-K	Si-K	Cl-K	K-K	Ca-K	Ti-K	Fe-K
**C7-03(1)_pt1**	+/-0.58	+/-0.10	+/-0.16	+/-0.19	+/-0.24	+/-0.07	+/-0.22		+/-0.21	+/-0.65
**C7-03(1)_pt2**	+/-0.60	+/-0.13	+/-0.13	+/-0.21	+/-0.33			+/-0.17		+/-0.26
**C7-03(1)_pt3**	+/-0.87	+/-0.19	+/-0.14	+/-0.26	+/-0.41		+/-0.15	+/-0.15		+/-0.74

Atom %

	O-K	Na-K	Mg-K	Al-K	Si-K	Cl-K	K-K	Ca-K	Ti-K	Fe-K
**C7-03(1)_pt1**	31.88	1.27	7.13	10.08	25.82	0.45	8.17		2.08	13.12
**C7-03(1)_pt2**	38.40	2.49	2.90	13.15	40.45			1.03		1.58
**C7-03(1)_pt3**	48.10	3.36	1.37	8.91	33.79		2.07	0.80		1.60

Atom % Error (+/- 1 Sigma)

	O-K	Na-K	Mg-K	Al-K	Si-K	Cl-K	K-K	Ca-K	Ti-K	Fe-K
**C7-03(1)_pt1**	+/-1.05	+/-0.12	+/-0.19	+/-0.20	+/-0.24	+/-0.06	+/-0.16		+/-0.13	+/-0.34
**C7-03(1)_pt2**	+/-0.89	+/-0.13	+/-0.13	+/-0.19	+/-0.28			+/-0.10		+/-0.11
**C7-03(1)_pt3**	+/-1.24	+/-0.19	+/-0.13	+/-0.22	+/-0.33		+/-0.09	+/-0.08		+/-0.30

Formula

	O-K	Na-K	Mg-K	Al-K	Si-K	Cl-K	K-K	Ca-K	Ti-K	Fe-K
**C7-03(1)_pt1**	O	Na	Mg	Al	Si	Cl	K		Ti	Fe
**C7-03(1)_pt2**	O	Na	Mg	Al	Si			Ca		Fe
**C7-03(1)_pt3**	O	Na	Mg	Al	Si		K	Ca		Fe

Compound %

	O	Na	Mg	Al	Si	Cl	K	Ca	Ti	Fe
**C7-03(1)_pt1**	17.72	1.01	6.03	9.45	25.20	0.56	11.10		3.46	25.46
**C7-03(1)_pt2**	26.01	2.42	2.98	15.02	48.09			1.74		3.73
**C7-03(1)_pt3**	33.87	3.40	1.46	10.58	41.77		3.56	1.41		3.94

Cations

	O-K	Na-K	Mg-K	Al-K	Si-K	Cl-K	K-K	Ca-K	Ti-K	Fe-K
**C7-03(1)_pt1**	0.00	0.00	0.00	0.00	0.00	0.00	0.00		0.00	0.00
**C7-03(1)_pt2**	0.00	0.00	0.00	0.00	0.00			0.00		0.00
**C7-03(1)_pt3**	0.00	0.00	0.00	0.00	0.00		0.00	0.00		0.00
